# Understanding Heterogeneity in the Impact of National Neglected Tropical Disease Control Programmes: Evidence from School-Based Deworming in Kenya

**DOI:** 10.1371/journal.pntd.0004108

**Published:** 2015-09-30

**Authors:** Birgit Nikolay, Charles S. Mwandawiro, Jimmy H. Kihara, Collins Okoyo, Jorge Cano, Mariam T. Mwanje, Hadley Sultani, Dorcas Alusala, Hugo C. Turner, Caroline Teti, Josh Garn, Matthew C. Freeman, Elizabeth Allen, Roy M. Anderson, Rachel L. Pullan, Sammy M. Njenga, Simon J. Brooker

**Affiliations:** 1 Faculty of Infectious and Tropical Diseases, London School of Hygiene & Tropical Medicine, London, United Kingdom; 2 Eastern and Southern Africa Centre of International Parasite Control, Kenya Medical Research Institute, Nairobi, Kenya; 3 Neglected Tropical Diseases Unit, Division of Communicable Disease Prevention and Control, Ministry of Health, Nairobi, Kenya; 4 London Centre for Neglected Tropical Disease Research, London, United Kingdom; 5 Department of Infectious Disease Epidemiology, School of Public Health, Faculty of Medicine, St Marys Campus, Imperial College London, London, United Kingdom; 6 Evidence Action, Nairobi, Kenya; 7 Department of Environmental Health, Rollins School of Public Health, Emory University, Atlanta, Georgia, United States of America; 8 Department of Medical Statistics, London School of Hygiene & Tropical Medicine, London, United Kingdom; Ministry of Health, UGANDA

## Abstract

**Background:**

The implementation of soil-transmitted helminth (STH) treatment programmes occurs in varied environmental, social and economic contexts. Programme impact will be influenced by factors that affect the reduction in the prevalence and intensity of infections following treatment, as well as the subsequent rate of reinfection. To better understand the heterogeneity of programme impact and its underlying reasons, we investigated the influence of contextual factors on reduction in STH infection as part of the national school based deworming (SBD) programme in Kenya.

**Materials and Methods:**

Data on the prevalence and intensity of infection were collected within the monitoring and evaluation component of the SBD programme at baseline and after delivery of two annual treatment rounds in 153 schools in western Kenya. Using a framework that considers STH epidemiology and transmission dynamics, capacity to deliver treatment, operational feasibility and financial capacity, data were assembled at both school and district (county) levels. Geographic heterogeneity of programme impact was assessed by descriptive and spatial analyses. Factors associated with absolute reductions of *Ascaris lumbricoides* and hookworm infection prevalence and intensity were identified using mixed effects linear regression modelling adjusting for baseline infection levels.

**Principal Findings:**

The reduction in prevalence and intensity of *A*. *lumbricoides* and hookworms varied significantly by county and within counties by school. Multivariable analysis of factors associated with programme impact showed that absolute *A*. *lumbricoides* reductions varied by environmental conditions and access to improved sanitation at schools or within the community. Larger reduction in prevalence and intensity of hookworms were found in schools located within areas with higher community level access to improved sanitation and within counties with higher economic and health service delivery indicator scores.

**Conclusions:**

The study identifies factors associated with the impact of school-based deworming and in particular highlights how access to water, sanitation and hygiene and environmental conditions influence the impact of deworming programmes.

## Author Summary

Most countries with endemic soil-transmitted helminth (STH) infections have started implementing deworming programmes in recent years. However, the achievable impact on the prevalence and intensity of infections will depend on the socioeconomic and environmental context in which the programme is implemented. We use a previously developed framework that considers the epidemiology of STH transmission, capacity to deliver treatment, operational and financial feasibility, to investigate reasons for the observed within-country variation of deworming programme impact based on data from the national school based deworming programme in Kenya. The study demonstrates that programme impact varied markedly within the country and that reductions in STH infection were associated with levels of access to water, sanitation and hygiene (at schools or within communities). The described framework can help to identify areas where lower programme impact can be expected, and also to determine which additional interventions should be implemented in support of the deworming efforts.

## Introduction

Soil transmitted helminths (STH: *Ascaris lumbricoides*, *Trichuris trichiura*, and the hookworms *Necator americanus* and *Ancylostoma duodenale*) are among the diseases classified by the World Health Organization as neglected tropical diseases (NTDs) [1]. STHs are endemic in 166 countries worldwide [2] and the majority of these countries are now implementing mass drug administration (MDA) programmes, either through school-based deworming (SBD) or lymphatic filariasis control programmes [3]. However, progress in ensuring treatment is targeted to at-risk communities has been highly variable [3] and coverage has not always been coincidental with patterns of transmission intensity. Where scaling-up of treatment has happened, it has occurred in a heterogeneous social and economic environment, such that the impact of STH treatment programmes will vary according to context.

The impact of STH treatment programmes is known to be influenced by a variety of different factors. Models of the transmission dynamics of STH indicate that reduction in the prevalence and intensity of infection following treatment, as well as the subsequent rate of reinfection, is influenced by the underlying intensity of transmission (as estimated by the basic reproductive number, R_0_), the efficacy of the drugs used, and the proportion of the overall population treated [4,5]. In turn, the intensity of transmission will be influenced by climatic factors that determine the development and survival of free-living stages in the external environment [6] and by levels of water, sanitation and hygienic behaviour [7] that determine the rate of exposure to ova and larvae. Drug efficacy of available anthelmintics is known to vary by STH species [8], such that the impact of treatment will also be influenced by the relative prevalences of the different STH species and the drugs used. Finally, the treatment coverage achieved by national deworming programmes will depend on the broader context of the programme in terms of local infrastructure and governance [9]. There is however limited evidence on the role of the above factors in determining the impact of STH treatment in the context of at-scale, national control programmes.

The specific aims of this study are to (i) describe the heterogeneity in impact of a national SBD programme and (ii) identify factors associated with the impact of the programme. We investigate the influence of these different contextual factors on the impact of SBD, using data from a national programme in Kenya.

## Materials and Methods

### The Kenya school-based deworming programme

Kenya implemented a national school-based deworming programme in 2009, with countrywide roll-out in 2012. Launched jointly by the ministries of health and education, the programme aims to deworm all school children living in sub-counties at high risk of STH infection and schistosomiasis over five years (2012–2017) [10]. To enable a targeted delivery of treatment, geographic areas with children requiring MDA were initially identified based on historical survey data and socio-environmental predictive risk models [11]. During the first two years of the programme, an estimated 6.4 million treatment doses were delivered yearly in 66 sub-counties with high STH endemicity in Western, Nyanza, Rift Valley and Coast regions [12].

The process and impact of the programme is evaluated through an independent monitoring and evaluation (M&E) programme, conducted by the Kenya Medical Research Institute, as described elsewhere [10]. The current analysis focuses on 153 schools surveyed in western Kenya at baseline (January to April 2012) and in year three of the programme (March to June 2014) following two rounds of annual MDA activities. The most recent deworming round was 7–13 months prior (median 9 months) to follow up. Surveys were repeat cross-sectional at selected schools, such that a random sample of children was selected at each survey. For each survey, approximately 100 children from each participating school were randomly selected (18 children from each of primary school classes 2–6 and ECD (early child development)) and asked to provide a stool sample which was examined in duplicate smears using the Kato-Katz method [13].

### Analysis framework

**Fig 1:**
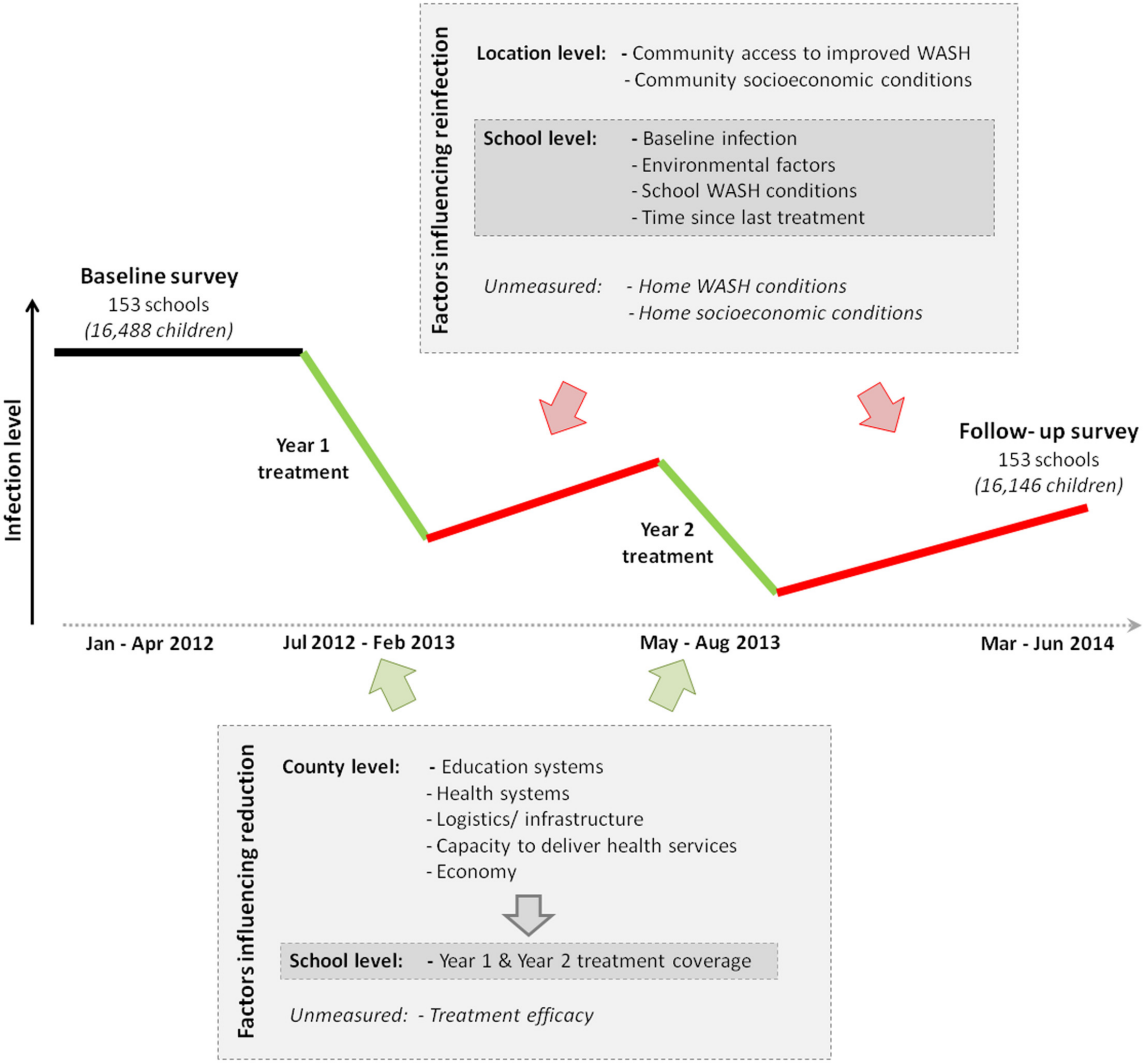
Analysis framework. The observed impact of the deworming programme between baseline and follow-up assessment is determined by immediate reductions in infections after year one and two treatment delivery followed by reinfections between the treatments. Immediate infection reductions are likely to be influenced by the treatment coverage, which itself can be influenced by the broader context of the programme, such as quality of education and health systems, infrastructure and capacity to deliver health services and the economic situation. The rate of reinfection is likely to be influenced by general risk factors for STH infections such as environmental conditions, water, sanitation, and hygiene (WASH) and socioeconomic conditions and baseline infection levels.

The observable impact of the school-based deworming programme between baseline (2012) and follow up assessment (2014) can be interpreted in terms of two processes, namely (i) immediate reductions in infections following treatment in year one and two, based on the efficacy of the deworming drugs and coverage of drug distribution and (ii) the rate of reinfection between the treatments (Fig 1). Analysis is based on a framework that identifies the factors associated with a successful STH control programme, including the political and economic context, country health and education systems, the inputs and outputs of the STH control programme, and the underlying epidemiology of infection [9]. The framework includes three major factors which influence programme success: (i) STH epidemiology which determines the rate of reinfection after treatment delivery, (ii) capacity to deliver treatment to the targeted population assessed through existing health and educational infrastructures, (iii) operational feasibility of the deworming programme determined by political commitment and governance, and financial capacity to ensure sustained infection control (Table A in S1 Text). In the present analysis, we identified and assembled relevant indicator data at the sub-national level in Kenya (Fig 1).

### Data and data sources

#### Administrative boundaries

The administrative boundaries in Kenya changed in 2013 and are now divided into eight regions (formerly referred to as provinces), which are divided into 47 counties and further into sub-counties (n = 290), wards and sub-wards. Wards often share common boundaries with the previously defined locations.

#### School level treatment coverage

Information on treatment coverage, as reported by the schools, is routinely recorded within the national deworming programme and was obtained at the school level from Evidence Action, an international non-governmental organization, who provides technical support to the government programme. The reporting of treatment coverage follows a “cascade system,” meaning that for each school, a school deworming summary form is completed by the head teacher including information on school name and location, date of deworming and the number of children enrolled and treated in each class. The completed form is subsequently sent to the area education officer who summarises the data by ward and sends it to the sub-county director of education where treatment data are summarised by sub-county.

#### School level water, sanitation and hygiene (WASH) indicators

WASH and socioeconomic conditions may influence the reinfection rate after treatment, as limited access to appropriate sanitation increases exposure of children to STH infectious stages [7]. Basic information on access to WASH at schools was collected during the baseline survey by interviewing the head teacher or deputy head teacher and by visual inspection (Table B in S1 Text). Data were missing for the following variables: availability of hand washing facility (four schools); water source (five schools); and health programme (four schools). Additionally, information was missing on the school sanitation type (five schools), proportion of clean toilets (six schools) and the number of children per toilet (five schools). The geographic distribution of school sanitation facility types is shown in Fig A in S1 Text.

#### School level environmental data

Environmental conditions are known to influence the survival of STH free-living stages, and therefore the transmission success of STHs. They include factors that are related to temperature and humidity [6,11,14]. Estimates of land surface temperature (LST), aridity index (AI), enhanced vegetation index (EVI), and population density were documented for each school and a buffer of 1km around each school, with data averaged over the array of estimates (Table B in S1 Text) [15–17].

#### Location level community WASH and socioeconomic data

Information on community access to WASH as well as socioeconomic indicators was derived from the 2009 Kenya Population and Housing Census [18] and aggregated at location level—typically a population of 7,000–25,000 (Table B in S1 Text). The previously defined locations, which were in place during the 2009 census, correspond roughly to the newly defined wards in Kenya. Aggregates were generated separately for rural, urban and peri-urban areas and matched to school locations accordingly, where possible. The geographic distribution of community level access to improved sanitation is shown in Fig A in S1 Text.

#### County level infrastructure, economy, education and health system data

County variables were selected based on the assessment of STH elimination feasibility framework described in Brooker *et al*. [9] and were representative of the following domains: wider health systems; wider education systems; delivery platforms; logistics/infrastructure; and economy. Data on county characteristics were derived from the Open Kenya database [19] and other open access resources [20,21] (see Table C in S1 Text for details). Variables within each indicator group were combined using principal component analysis (PCA) and the first component of each indicator group was used for further analysis. Details on included variables, sources of data and component loadings are provided in Table C in S1 Text.

### Infection levels at baseline and follow-up

Infection data based on duplicate Kato-Katz smears for each child were obtained from cross-sectional surveys at baseline (year one) and follow-up (year three) as previously described. Statistical analysis was carried out at the school level as children were not followed up individually. We were interested in the impact of the control programme on both prevalence and arithmetic average intensity of infections. Summary statistics on both outcomes were calculated by survey for STH combined, and each species separately as described below.

For prevalence of infection, the outcome was specified as the number of positive children in each school per survey round with the number of children tested in a school as the number of trials. Point estimates and confidence intervals (95% CIs) for prevalence were obtained using school level binomial regression models, adjusting for clustering within counties. For average intensity of infections, we used the count of eggs in children’s stool as a proxy for worm intensity [22]. The outcome was specified as total egg counts in each school (the sum of eggs counted on two slides per child within a school) per survey round and the number of children surveyed in a school as the number of trials. Point estimates and 95% CIs of the average intensity of infection were obtained using school level negative binomial regression models adjusting for clustering within counties. Intensity summary statistics were transformed to average eggs per gram faeces (epg) for presentational purposes. Additionally, intensity of infection was classified according to WHO guidelines as ≥ 5,000 epg for *A*. *lumbricoides*, ≥2,000 epg for hookworm and ≥ 1,000 epg for *T*. *trichiura* to obtain the prevalence of moderate-heavy infections [22]. Data were analysed using Stata 13 (College Station, TX, USA).

### Assessment of programme impact

Programme impact on prevalence of infection was quantified using school level mixed effects logistic regression models with a random intercept at the county level. The outcome was again specified as the number of positive children in each school and the number of children tested in a school as the number of trials. The reduction in average intensity of infection was estimated using school level negative binomial regression models with a random intercept at county level. The outcome was specified as the total school egg counts with the number of surveyed children as the number of trials. For both logistic and negative binomial models, baseline and follow-up infections were treated as repeated measures outcomes and survey round was included in all models as a covariate.

Maps were created for the relative reduction (percentage reduction) of prevalence and intensity by school and county using ArcGIS Desktop 10 (Redlands, CA, USA). Graphs were created using the ggplot package in R [23].

### Factors associated with programme impact

Patterns in the two-year impact of the deworming programme were investigated in relation to treatment coverage, baseline infection, WASH and environmental characteristics of schools or the wider environment using baseline and follow-up parasitological data collected in 153 schools. A detailed description of all considered variables is provided in Tables B and C in S1 Text and correlations of variables are presented in Table D in S1 Text. The analysis was performed separately for changes in prevalence and average intensity of *A*. *lumbricoides* and hookworm infections. *T*. *trichiura* was not considered for this analysis as baseline infections were low.

As we were interested in factors directly associated with changes in infection levels, the outcome variables were defined at school level as absolute difference in prevalence (follow-up prevalence minus baseline prevalence) and absolute difference in arithmetic average school epg (follow-up average epg minus baseline average epg). For both outcomes, negative values indicate a decrease in infections at school level and positive values an increase. We then modelled the association of outcome variables with school, location, and county characteristics using multivariable mixed effects linear regression models with a random intercept for counties. A location level random intercept was not included in the model, as only a few schools clustered within a location. As absolute changes are highly dependent on baseline infection levels (a greater absolute reduction can be achieved where baseline infections are higher), models were adjusted by default for the relevant species specific school baseline prevalence or average intensity of infection (epg), respectively. Due to evidence of non-normality in the continuous outcome measures, non-parametric bootstrapping was used to estimate bias corrected 95% CIs [24].

Associations of variables with absolute changes in prevalence and average epgs were first tested in univariable analysis and variables were considered for further investigation in multivariable analysis when 95% CIs of coefficients did not include zero. To avoid collinearity in multivariable models, the covariance of selected variables was investigated, however no strong correlation (r≥0.70) was observed. Multivariable mixed effects linear regression models were developed using a backwards approach, where all variables identified in the univariable analysis were included and eliminated one at a time until a parsimonious model was obtained. The final model included only variables with coefficient 95% CIs that did not include zero. Schools with missing data were excluded from the analysis whenever the missing variable was included in the models. A sensitivity analysis was performed replacing missing values as minimum or maximum observed values to assess the influence of missing data on the analysis.

The spatial correlation of absolute prevalence and epg reductions (adjusted for baseline infection) was investigated by semivariogram analysis implemented in the geoR package in R [23]. Semivariograms were plotted for normal score transformed residuals of linear regression models adjusting for baseline infection only, as well as for residuals of multivariable models adjusting for all associated variables. Heterogeneity in the spatial distribution of school infection reductions was assessed by computing empirical variogram envelops by permutations of the data values on the spatial locations (100 simulations).

The underlying data of this article are provided in S1 File.

### Ethics statement

Ethical approval was obtained by the Kenya Medical Research Institute (KEMRI) Ethical Review Committee (SSC no. 2206). All data used were anonymised.

## Results

### Programme impact after two rounds of MDA delivery

**Fig 2:**
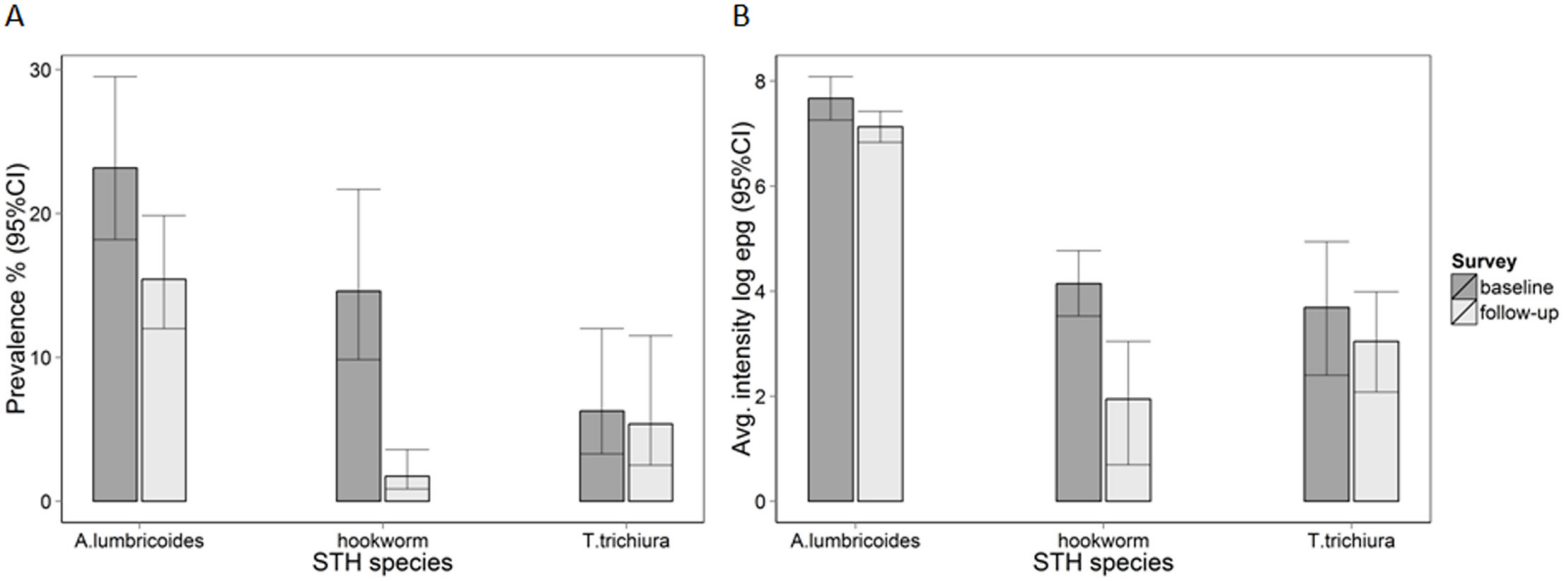
Prevalence (A) and average intensity of infection (natural-log scale) (B) by survey round and STH species. Infection levels were measured in 153 schools at baseline (2012) and follow-up (2014).

In the 153 schools surveyed at baseline in 2012, the combined STH prevalence was 34.8%, with *A*. *lumbricoides* most prevalent (23.2%), followed by hookworm (14.6%) and *T*. *trichiura* (6.3%). At follow-up, after two rounds of MDA, STH prevalence dropped to 19.7%; with prevalence decreasing to 15.4%, 1.7%, and 5.4% for *A*. *lumbricoides*, hookworms and *T*. *trichiura*, respectively (Fig 2 and Table 1). The mean intensity of *A*. *lumbricoides* infection fell significantly from 2,147 epg at baseline to 1,248 epg at follow-up and hookworm from 63 epg to 7 epg; mean *T*. *trichiura* intensity changed from 40 epg to 21 epg over the same time period, but the change was non-significant (Fig 2 and Table 1). The prevalence of moderate and heavy infection was reduced from 11.1% (95% CI: 7.8; 15.7) at baseline to 7.4% (95% CI: 5.6; 9.9) at follow-up (OR 0.63, 95% CI: 0.59; 0.68, p<0.001).

**Table 1:** Comparison of infection prevalence and average intensity at baseline (2012) and follow-up (2014) in 153 schools. Odds ratio (OR) and epg ratio were calculated using school level mixed effects logistic and negative binomial regression models, respectively, taking into account clustering by counties.

Species	Baseline prevalence/ mean epg (95% CI)	Follow-up prevalence/ mean epg (95% CI)	Absolute reduction (%/ epg)	Relative reduction (%)	OR/epg ratio (95% CI)	p-value^1^
**Prevalence of infection**						
STH combined	34.76 (29.91; 40.39)	19.68 (15.28; 25.34)	15.08	43.38	0.44 (0.42; 0.47)	<0.001
*A*. *lumbricoides*	23.17 (18.19; 29.52)	15.44 (12.00; 19.86)	7.73	33.36	0.59 (0.56; 0.62)	<0.001
Hookworm	14.61 (9.84; 21.69)	1.75 (0.85; 3.60)	12.86	88.02	0.10 (0.08; 0.11)	<0.001
*T*. *trichiura*	6.28 (3.29; 12.01)	5.38 (2.51; 11.51)	0.9	14.33	0.83 (0.75; 0.91)	<0.001
**Average intensity of infection**						
*A*. *lumbricoides*	2,147 (1,420; 3,246)	1,248 (929; 1,675)	899	41.89	0.62 (0.47; 0.83)	0.002
Hookworm	63 (34; 118)	7 (2; 21)	56	88.92	0.06 (0.03; 0.10)	<0.001
*T*. *trichiura*	40 (11; 140)	21 (8; 54)	19	48.32	0.78 (0.46; 1.33)	0.364

^1^ based on likelihood ratio test

**Fig 3:**
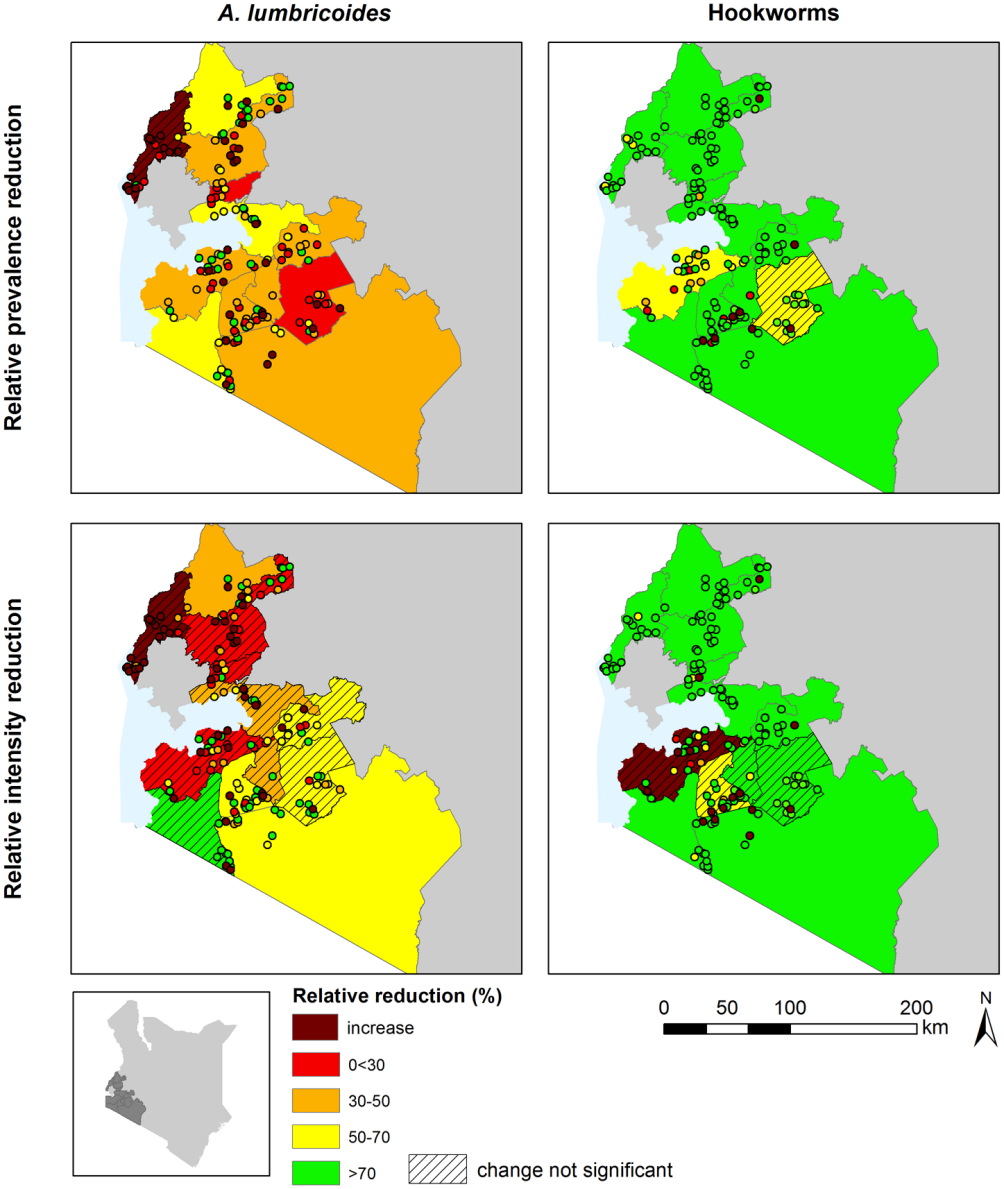
School and county reductions in *A*. *lumbricoides* and hookworm infections. The maps show the relative reduction in *A*. *lumbricoides* and hookworm prevalence and average intensity of infections surveyed in 153 schools at baseline (2012) and follow-up (2014). The statistical significance (p<0.05) of observed within county level changes was assessed by school level logistic and negative binomial regression analysis.

**Fig 4:**
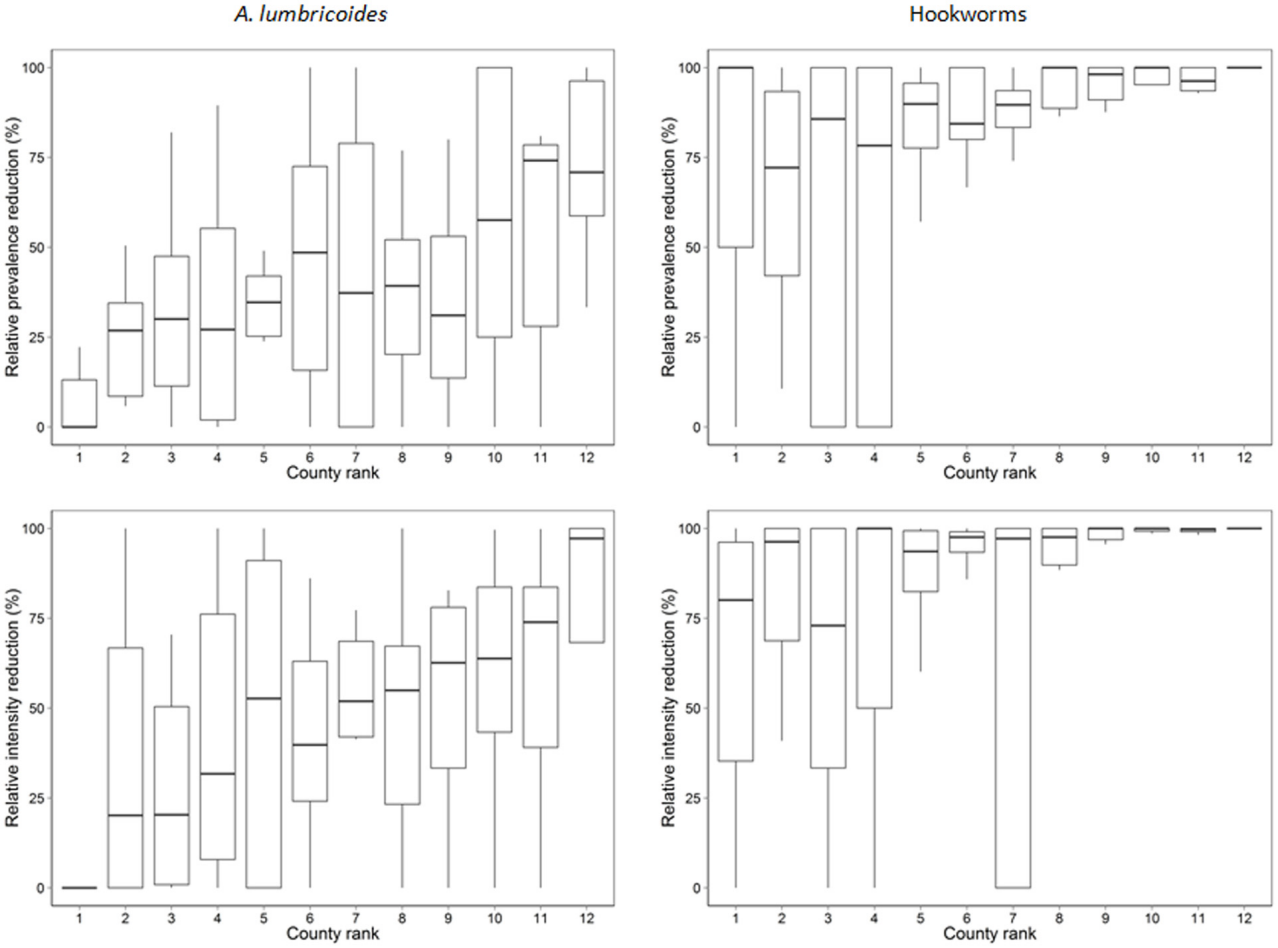
School heterogeneity in relative infection reductions within counties. Counties were ranked lowest to highest according to the relative reduction in prevalence and average intensity of infection. For hookworm, 13 schools with zero infections in both the baseline and follow-up surveys were excluded from the box-plot; schools with increase in infection are plotted as zero relative reduction.

The geographic heterogeneity in relative reductions between baseline and follow-up surveys of *A*. *lumbricoides* and hookworm infections is shown in Fig 3. Prevalence and intensity reductions varied significantly by county for *A*. *lumbricoides* and hookworm (county random effects, all p<0.001). The relative reduction of infections also varied within counties by school (Fig 4). This heterogeneity was overall more apparent for *A*. *lumbricoides* prevalence and intensity reductions than for hookworm. However, for hookworm, the heterogeneity was more pronounced in counties with lower overall reductions. An increase in infection (prevalence or intensity) between baseline and follow-up was observed in 54 and 15 schools for *A*. *lumbricoides* and hookworm, respectively. Thirteen schools had zero hookworm infections at baseline and follow-up. These schools were mainly located in Bomet County and in areas with low land surface temperature. Maps showing baseline and follow-up infections by school and county are provided in Fig B and Fig C in S1 Text.

#### Factors associated with programme impact

In univariable analysis, of the 27 considered variables, four variables were associated with absolute changes in *A*. *lumbricoides* prevalence and six with changes in hookworm prevalence (Table E in S1 Text). Five and eight variables were associated with changes in *A*. *lumbricoides* and hookworm intensities, respectively.

Results of the multivariable analysis of factors associated with programme impact are summarised in Table 2. The final multivariable models showed evidence for a larger reduction in the prevalence and intensity of *A*. *lumbricoides* among schools located in areas with higher mean land surface temperature. The impact of treatment on prevalence of *A*. *lumbricoides* was also greater among schools with ventilated improved pit (VIP) or waterborne sanitary facilities compared to ordinary pit latrines and smaller among schools surveyed 350–385 days after second MDA delivery compared to schools surveyed 209–250 days post MDA. The impact on *A*. *lumbricoides* intensity was greater in areas with higher vegetation index, in locations with medium community level access to improved sanitation (waterborne, VIP and covered pit latrines) and in counties with a high education indicator score. Reductions were lower in medium populated areas (indicative of a peri-urban environment), in schools with higher baseline prevalence, and in counties with higher health system indicator scores.

**Table 2:** Factors associated with programme impact measured as absolute change since baseline survey. A negative coefficient indicates a greater absolute reduction. Estimates were obtained by multivariable mixed effects linear regression analysis adjusting for baseline infections and with a random intercept for counties. Final models were additionally adjusted for variables indicated in italic; variables with 95% CIs not overlapping zero are indicated in bold. A detailed table summarising results for all investigated variables is provided in Table F in S1 Text.

		*A*. *lumbricoides*		Hookworm	
		Prevalence reduction^1^	Average epg reduction	Prevalence reduction^2^	Average epg reduction^2^
**Variable**	**Categories**	**Coefficient (95%CI)** ^3^	**Coefficient (95%CI)** ^3^	**Coefficient (95%CI)** ^3^	**Coefficient (95%CI)** ^3^
**LST**	<30°C	***base***	***base***	base	base
	30–35°C	***-3*.*76 (-6*.*85; 0*.*58)***	***-201*.*04 (-623*.*51; 353*.*47)***	0.13 (-0.61; 0.85)	-3.93 (-20.30; 8.09)
	≥35°C	***-7*.*60 (-11*.*55; -2*.*91)***	***-680*.*28 (-1*,*090*.*53; -144*.*63)***	-1.65 (-3.93; 1.04)	-14.23 (-39.71; 2.66)
**EVI**	<0.4	base	**base**	base	base
	≥0.4	-0.85 (-4.98; 2.70)	**-503.34 (-1437.99; -75.24)**	0.29 (-0.58; 1.53)	-0.40 (-12.16; 7.07)
**Population density (per100m^2^)**	<5	base	**base**	base	base
	5–10	3.61 (-0.01; 7.73)	**470.00 (188.08; 829.00)**	-0.63 (-1.37; 0.01)	-2.77 (-11.40; 0.58)
	≥10	2.97 (-3.64; 8.80)	**103.64 (-490.25; 903.07)**	0.25 (-0.89; 1.51)	10.63 (-0.99; 26.97)
**Baseline prevalence**	<20%	NA	**base**	NA	base
	20–40%		**137.90 (-224.24; 581.53)**		7.05 (-5.50; 18.29)
	≥40%		**614.58 (51.51; 1,395.64)**		-7.64 (-43.83; 13.82)
**Time since Y2 treatment**	<250 d	**base**	base	base	base
	250–300 d	**0.70 (-3.67; 5.06)**	123.08 (-500.60; 895.54)	-0.13 (-1.01; 0.69)	7.28 (-5.61; 25.72)
	300–350 d	**0.43 (-4.43; 4.61)**	43.71 (-456.76; 609.73)	-0.64 (-1.54; 0.13)	6.24 (-4.76; 18.76)
	≥350 d	**5.04 (0.38; 11.42)**	226.02 (-412.39; 1,059.72)	1.25 (-0.43; 3.21)	1.61 (-15.74; 14.21)
**Socioeconomic score**	<20	base	base	base	***base***
	≥20	-1.92 (-4.58; 1.47)	-272.76 (-616.92; 139.43)	-0.61 (-1.86; 0.52)	***-8*.*48 (-26*.*06; 0*.*00)***
**Access impr.sanitation(waterborne, VIP & covered pit)**	<50%	base	**base**	**base**	base
	50–75%	-1.87 (-5.08; 0.53)	**-338.26 (-702.91; -43.68)**	**0.38 (-0.89; 1.48)**	-6.55 (-25.53; 4.29)
	≥75%	-0.22 (-4.28; 3.81)	**-127.92 (-531.20; 334.91)**	**-1.31 (-2.77; -0.12)**	-4.17 (-21.85; 2.56)
**Access improved drinking water**	<50%	base	base	base	**base**
	50–75%	2.44 (-0.37; 7.45)	232.48 (-171.64; 748.06)	0.55 (-1.02; 1.87)	**9.78 (0.97; 23.58)**
	≥75%	-1.30 (-5.21; 2.37)	245.86 (-28.95; 936.26)	-0.51 (-1.70; 0.66)	**2.03 (-5.62; 11.12)**
**School water source**	piped	base	base	***base***	***base***
	borehole/well	5.54 (-0.09; 10.75)	231.02 (-239.11; 731.45)	***1*.*07 (-0*.*18; 2*.*55)***	***1*.*95 (-5*.*04; 8*.*56)***
	rain	1.50 (-3.48; 5.80)	-100.78 (-633.82; 329.13)	***1*.*05 (0*.*18; 2*.*23)***	***13*.*71 (0*.*35; 39*.*99)***
	river	3.08 (-1.76; 7.65)	138.20 (-425.96; 686.50)	***0*.*97 (0*.*03; 2*.*00)***	***1*.*94 (-5*.*52; 7*.*95)***
	others	-1.88 (-8.47; 4.42)	-486.92 (1,302.04; 97.47)	***0*.*11 (-2*.*03; 2*.*13)***	***-3*.*32 (-15*.*77; 8*.*68)***
**School sanitation**	pit latrine	***base***	base	base	base
	VIP& waterborne	***-3*.*53 (-7*.*41; -0*.*54)***	172.71 (-496.50; 141.08)	0.57 (-1.21; 2.31)	-9.17 (-31.50; 2.17)
**Children per toilet**	<25	base	Base	base	**base**
	25–50	-0.26 (-3.31; 3.68)	195.38 (-180.85; 669.40)	0.57 (-0.29; 1.53)	**3.89 (0.42; 8.84)**
	≥50	0.20 (-4.21; 4.35)	324.07 (-168.11; 839.34)	-0.47 (-1.82; 0.89)	**2.48 (-4.87; 13.62)**
**County education score**	1^st^ tertile	base	**base**	base	***base***
	2^nd^ tertile	-0.19 (-4.23; 3.22)	**219.50 (-281.22; 692.84)**	1.45 (-0.31; 3.32)	***12*.*73 (2*.*74; 32*.*00)***
	3^rd^ tertile	-2.87 (-6.64; 0.81)	**-676.58 (-1129.10; -246.78)**	0.16 (-1.14; 1.75)	***-3*.*95 (-17*.*51; 1*.*76)***
**County health system score**	1^st^ tertile	base	***base***	***base***	**base**
	2^nd^ tertile	3.66 (-1.04; 6.85)	***711*.*08 (294*.*72; 1175*.*92)***	***1*.*85 (0*.*79; 2*.*79)***	**23.71 (4.89; 58.48)**
	3^rd^ tertile	1.83 (-1.56; 4.98)	***387*.*42 (77*.*20; 750*.*22)***	***1*.*17 (0*.*09; 2*.*13)***	**22.94 (5.66; 58.45)**
**County health service delivery score**	1^st^ tertile	base	base	***base***	base
	2^nd^ tertile	-0.10 (-3.31; 3.27)	108.19 (168.70; 453.66)	***-1*.*63 (-2*.*87; -0*.*39)***	-0.42 (-6.42; 6.11)
	3^rd^ tertile	-1.53 (-6.23; 2.87)	235.99 (-238.77; 722.44)	***-1*.*33 (-2*.*63; -0*.*27)***	0.91 (-11.65; 8.48)
**County economy score**	1^st^ tertile	base	base	***base***	**base**
	2^nd^ tertile	-1.78 (-5.35; 1.70)	209.57 (-302.06; 677.31)	***-0*.*68 (-1*.*55; 0*.*22)***	**-13.39 (-37.08; -1.66)**
	3^rd^ tertile	-1.49 (-4.61; 1.62)	-126.97 (-501.71; 227.90)	***-1*.*23 (-1*.*97; -0*.*49)***	**-0.46 (-11.72; 8.89)**
**Random effect (sd)**	County	2.23 (0.00; 3.75)	274.23 (0.00; 541.04)	0.53 (0.00; 0.88)	0.00 (0.00; 4.95)

^1^6 schools with missing school sanitation information were excluded from final models

^2^5 schools with missing school water source information were excluded from final models

^3^Bias corrected 95% CI

Greater reductions in hookworm prevalence were associated with high community level access to improved sanitation, as well as county economy and health service delivery indicator scores. Reductions were smaller among schools with rain or river as main water source, and also in counties with a higher health system indicator score. The impact on hookworm intensity was greater among schools located in areas with higher socioeconomic scores and in counties with medium economic indicator scores. Smaller reductions were observed in schools with rain as main water-source and medium number of children per school toilet, in areas with medium access to improved drinking water, and counties with medium education and high health system scores. County level random effects were not significant in multivariable models.

Surprisingly, treatment coverage was not associated with programme impact, but treatment coverage was generally high (>90%) across the counties, especially in the second year of the programme. Where coverage varied by county, this was associated with the quality of education systems (Table J in S1 Text and Fig D in S1 Text). Detailed results for all investigated variables are shown in Table F in S1 Text. Results of the sensitivity analysis are presented in Tables G and H in S1 Text. Variables with 95% CIs close to zero were most sensitive to the imputation of both low and high missing values, probably due to inclusion of schools which were otherwise excluded from final models. For comparison, factors associated with baseline infection are presented in Table I in S1 Text.

**Fig 5:**
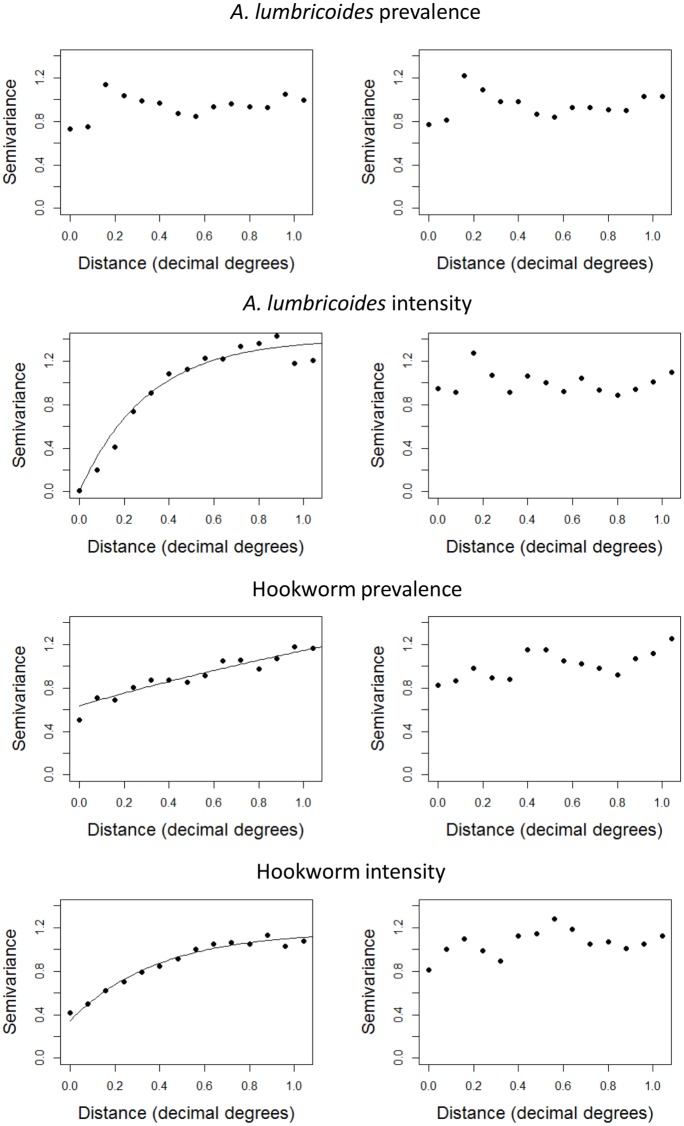
Omnidirectional semivariogram analysis of absolute reduction estimates adjusted for baseline infection (left panel) and additionally for other associated variables (right panel). Values were obtained from normal score transformation of linear regression residuals. Exponential models were fitted to the empirical semivariogram.

Spatial analysis of observed absolute reductions in prevalence and intensity (adjusted for baseline infection levels) indicated marked spatial dependency of reductions in *A*. *lumbricoides* and hookworm intensity (Fig 5). However, after removal of large scale trends by adjusting for associated factors in multivariable models, no clear spatial dependence was observed for the reduction in any of the infections (Fig 5). Comparing empirical semivariograms to the computed random permutation envelopes showed no evidence of spatial clustering in the residuals.

## Discussion

With the current upscaling of school-based deworming it is important to understand the variation of programme impact between and within countries, and the factors associated with the observed heterogeneity in impact. Here we show that the impact of STH treatment delivered as part of the Kenyan national SBD programme varied geographically and by STH species. Furthermore, guided by an analytical framework that evaluates the effectiveness of STH control programmes [9], we demonstrate that such heterogeneity was associated with variation in access to WASH, socioeconomic conditions, climate, and time since treatment.

Our findings reveal substantial variations in programmatic effectiveness between counties in Kenya. Comparison with previous evaluations of national control programmes in sub-Saharan Africa and Asia also shows that impact differs between countries (Table 3). Furthermore, the greater programme impact against hookworm compared to *A*. *lumbricoides* or *T*. *trichiura* is consistent with a meta-analysis of soil-transmitted helminth reinfections after treatment, which showed that *A*. *lumbricoides* and *T*. *trichiura* infection levels were comparable between baseline assessment and 12 months after treatment, while levels of hookworm infection were reduced by approximately half [25]. Mathematical modelling of STH transmission dynamics highlights that reduction in levels of infection and rates of reinfection post treatment are strongly influenced by species-specific characteristics, including the life expectancy of the adult worm, the underlying intensity of transmission (as measured by R_0_), environmental conditions that influence the development and survival of free-living stages in the external environment, the relative rate of infection of different age groups, and drug efficacy [26]. The slower rate of reinfection for hookworm is to be expected on the basis of the longer life expectancy of this parasite by comparison with *A*. *lumbricoides* [4,5]. The adult worm life expectancy sets the generation time of reinfection post treatment, with longer life expectancies resulting in lower rates of reinfection. Baseline infection levels were generally lower for hookworms than for *A*. *lumbricoides* indicating less contamination of the environment with infective larvae and, more generally, lower R_0_ values. Third stage hookworm larvae have a significantly shorter life expectancy of approximately 3–10 days compared to *A*. *lumbricoides* eggs with an infective period of up to several months [27–31]. Therefore, the environment can recover more quickly from hookworm contamination than from *A*. *lumbricoides*. Species-specific differences in drug efficacy are unlikely to explain observed species differences in impact since albendazole has high efficacy against both *A*. *lumbricoides* and hookworm [32]. However, the low observed programme impact on *T*. *trichiura* may be explained by the known low efficacy of albendazole against *T*. *trichiura*.

**Table 3:** Reported impact of MDA in national deworming programmes. Intervention groups are communities (individuals >2 years of age), school-aged children (SAC), and pre school-aged children (PSAC) and anthelmintics types are albendazole (ALB), mebendazole (MEB), praziquantel (PZQ), and diethylcarbamazine (DEC).

Country	Years of MDA	MDA frequency	Intervention group	Type of anthelmintic	Relative prevalence reduction (%)			Reference
					*A*. *lumbricoides*	Hookworm	*T*. *trichiura*	
Uganda	2	annual	SAC + community in selected locations	ALB+PZQ	78.5	79.0	27.3	[33]
Tanzania	1	annual	SAC	ALB+PZQ	-	19.7	-	[34]
China	2	annual	community	ALB	4.0	93.3	27.7	[35]
	2	bi-annual	community	ALB	50.1	84.3	19.7	
	2	bi-annual^1^	community	ALB	75.0	72.7	41.5	
India	2	annual	community	DEC+ALB	82.7	69.1	62.5	[36]
Indonesia	5	annual	community	DEC+ALB	20.6	85.7	81.8	[37]
Laos	1	bi-annual	PSAC+ SAC	MEB	66.7	increase	26.2	[38]
Myanmar	7	bi-annual	PSAC + SAC	ALB	88.0	95.4	67.7	[39]
Sri Lanka	4	annual	community	DEC+ALB	14.9	50.0	increase	[40]

^1^additional intervention: construction of latrines

The results show that observed reductions in the prevalence and intensity of both *A*. *lumbricoides* and hookworm varied markedly between counties and between schools within counties. The treatment coverage was generally high and as such there was no evidence for association of treatment coverage with programme impact (Table F in S1 Text). It is likely therefore that the observed differences in programme impact are due to geographic variations in the rates of reinfection between treatment rounds which are determined by the basic reproductive number of the parasites in different village locations. Previous studies have shown that the availability of improved sanitary conditions can enhance the impact of deworming programmes by limiting exposure of children to STH infectious stages in the external environment (and hence lowering the effective reproductive number) [41,42,7,43]–a finding confirmed in the present study. The present study also showed that reductions in *A*. *lumbricoides* prevalence were associated with access to VIP or waterborne latrines at schools, whilst reductions in *A*. *lumbricoides* intensity and hookworm prevalence were associated with access to improved home sanitation (waterborne, VIP and covered pit latrines) measured at community level. Similar patterns have also been observed during the baseline assessment of risk factors of infections, where, among others factors, higher *A*. *lumbricoides* infections were associated with poor school sanitation, and higher hookworm infection levels with poor home sanitation [44]. Moreover, a randomized-controlled trial of a school WASH programme in western Kenya showed that school based interventions reduced *A*. *lumricoides* reinfection rates, but not those for hookworms and *T*. *trichiura* [45]. Therefore, to achieve a major impact on STH transmission in Kenya, additional hygiene and sanitation programmes may be needed [46]. However, the cost-effectiveness of such additional interventions on infection levels needs to be carefully established, as these interventions are expensive and their effectiveness require a long time horizon and depend on cultural factors.

Some of the observed associations in this study seem not to be of causal nature, however, investigated variables may provide an indication for the general environment where children live. For example, a smaller reduction in *A*. *lumbricoides* intensity was observed in areas with medium population density, whereas high population density had no such effect. It may be that a peri-urban environment with less developed structures than an urban area and with people living in closer proximity than in a rural area increases the risk of reinfection. Similarly, the associations of reductions with several of the county indicator scores can be seen in such a context. However, to identify specific characteristics of these settings that may directly lead to higher reinfection rates, more detailed studies on the interrelatedness of WASH and housing conditions are required. Moreover, even though observational studies can help to identify candidate interventions, randomized controlled trials will be needed to ascertain a causal effect of specific interventions and select those that can effectively improve the impact of deworming programmes.

The variation in associations with prevalence and intensity reductions of the same species were not unexpected. Prevalence and intensity of infections measure two different characteristics of transmission levels and while intensity provides an indication of transmission intensity and disease burden, prevalence of infections with any intensity reveals the overall exposure to infectious agents in the population [47]. The goal of a deworming programme may transition from the reduction of disease burden to elimination of infections once transmission levels were lowered substantially [9]. Therefore, the results for prevalence and intensity reductions should be regarded as complementary to improve the impact of deworming on both measures.

The school treatment coverage was generally high in both years; especially in year two where all but three of the surveyed schools achieved treatment coverage >80%. Treatment coverage was higher in schools in counties with a better quality of education systems (Table J in S1 Text). Although this is not unexpected for a school-based deworming programme, the analysis emphasizes that a good school infrastructure is a prerequisite for the successful implementation of a school-based deworming programme [9]. Interestingly, other socio-economic and governance related indicators assessed were not associated with treatment coverage.

The study has several limitations, especially in relation to the quality and completeness of the collected data. Owing to the practicalities of conducting monitoring and evaluation on a large scale, the same school children were not followed up between baseline and follow-up survey (a cohort monitoring approach). However, pupils were randomly sampled and therefore should be representative of the school [10]. The results of parasitological surveys can also be influenced by the diagnostic accuracy of the Kato-Katz method, which generally has low sensitivity in low intensity settings and can therefore lead to an underestimation of infection levels [48]. Infection levels were assessed only in school aged children and school-based surveys do not accurately capture what is happening in the entire community, especially for hookworm infections that are generally of higher intensity in adults by comparison with children [26,49]. School WASH indicators were collected during baseline surveys by completion of a single questionnaire by the school. Schools may have mixed latrine types which would not have been captured by the administered questionnaire and the experience of field officers in the correct identification of latrine types may vary. Indeed, latrines may have been classified as VIP latrines in the presence of a pipe but may not have the other characteristics required by this classification. Community level access to WASH was obtained from census data; however, detailed questionnaires about home WASH conditions of surveyed children might have revealed additional associations. Treatment coverage was self-reported at school level and the accuracy may therefore also vary. Similarly, the quality of available county indicator data is unknown. Finally, increased awareness of STH infections resulting from taking part in the NSBDP might in turn lead to improvements in hygiene behaviours, reducing transmission risk.

In conclusion, the reported study showed that the impact of the national SBD programme in Kenya varied geographically and by STH species and that much of this variation could be captured using a framework that considers the epidemiology of STH transmission, capacity to deliver treatment and programmatic feasibility. Impact was associated with WASH access in schools and communities and other socioeconomic conditions as well as local climatic characteristics. An increased understanding of the variation in impact of STH control programmes, and factors associated with such heterogeneity, will help identify areas with lower expected impact, where enhanced efforts to expand treatment and improve WASH are required.

## Supporting Information

S1 Text Supplementary material. The document contains additional information, tables and figures as listed below. **Table A.** Domains and indicators of the STH elimination feasibility framework. **Table B.** School and location level components included in the analysis. **Table C.** County level indicators and components. **Table D.** Pair wise correlation matrix of components. **Table E.** Results of the univariable analysis of factors associated with programme impact. **Table F.** Detailed results of the multivariable analysis of factors associated with programme impact. **Table G.** Results of the sensitivity analysis with low imputed missing values. **Table F.** Results of the sensitivity analysis with high imputed missing values. **Table I.** Factors associated with baseline infection. **Table J.** Factors associated with treatment coverage. **Fig A.** Geographic distribution of WASH indicators. **Fig B.** Geographic distribution of *A*. *lumbricoides* baseline and follow up infections. **Fig C.** Geographic distribution of hookworm baseline and follow up infections. **Fig D.** Geographic heterogeneity of treatment coverage.
(DOCX)

S1 File 153 schools dataset. The file contains the underlying data.
(XLS)
