# Supplementary material for: Understanding Heterogeneity in the Impact of National Neglected Tropical Disease Control Programmes: Evidence from School-Based Deworming in Kenya
Source: PLoS Negl Trop Dis. 2015 Sep 30;9(9):e0004108. doi: 10.1371/journal.pntd.0004108 (PMC4589351; doi:10.1371/journal.pntd.0004108)
Supplement: S1 Text — The document contains additional information, tables and figures as listed below. Table A. Domains and indicators of the STH elimination feasibility framework. Table B. School and location level components included in the analysis. Table C. County level indicators and components. Table D. Pair wise correlation matrix of components. Table E. Results of the univariable analysis of factors associated with programme impact. Table F. Detailed results of the multivariable analysis of factors associated with programme impact. Table G. Results of the sensitivity analysis with low imputed missing values. Table F. Results of the sensitivity analysis with high imputed missing values. Table I. Factors associated with baseline infection. Table J. Factors associated with treatment coverage. Fig A. Geographic distribution of WASH indicators. Fig B. Geographic distribution of A. lumbricoides baseline and follow up infections. Fig C. Geographic distribution of hookworm baseline and follow up infections. Fig D. Geographic heterogeneity of treatment coverage. (DOCX) [file pntd.0004108.s001.docx]

**Supplementary information to**

**“Understanding heterogeneity in the impact of national neglected tropical disease control programmes: evidence from school-based deworming in Kenya”**

Contents

[**1. Determinants of programme impact- additional information** 1](#_Toc426462846)

[**1.1. Detailed information on used indicators and components** 1](#_Toc426462847)

[**1.2. Correlation among covariates** 5](#_Toc426462848)

[**1.3. Geographical distribution of WASH access** 6](#_Toc426462849)

[**1.4. Geographical distribution of baseline and follow-up infection levels** 6](#_Toc426462850)

[**1.5. Additional results- Univariable and multivariable analysis** 8](#_Toc426462851)

[**1.6. Missing data sensitivity analysis** 12](#_Toc426462852)

[**2. Factors associated with baseline infections** 14](#_Toc426462853)

[**3. Determinants of treatment coverage** 15](#_Toc426462854)

[**References** 17](#_Toc426462855)

**1. Determinants of programme impact- additional information**

**1.1. Detailed information on used indicators and components**

The successful implementation of a deworming programme is strongly dependent on its context. A previous study established a framework to assess STH elimination feasibility, ranking countries according to predefined indicators, some of which are expected to impact the capability of a country to deliver mass drug administration (MDA) (Table A) [[1](#_ENREF_1)]. Even though these criteria were established at country scale, similar relationships can be expected at smaller geographical scales such as counties, locations and schools.

To investigate whether these predefined indicators allow explaining the observed heterogeneity in programme impact, we selected variables representative of the indicators intensity of transmission and environmental exposure at school and location level (Table B). Included intensity of transmission components were environmental suitability of transmission (school level) and baseline infections (school level); included environmental exposure components were community access to water, sanitation, and hygiene (WASH) and socioeconomic conditions (location level) and access to WASH at schools (school level). Details and sources of components are summarised in Table B.

We further identified county level variables related to the five indicators health systems, education systems, logistics/ infrastructure, and economy as summarised in Table C. Variables were obtained mainly from the Kenya County Health Fact Sheets [[2](#_ENREF_2)] and Kenya County Fact Sheets (accessed via Kenya open data [[3](#_ENREF_3)]) (see Table C for details). County level indicators were derived from single variables using principal component analysis (PCA), where the PCA scores for the first component of each indicator were predicted and used for further analysis. The PCA component loadings are summarised in Table C.

| **Domain** | **Indicator** | | **Component** | |
| --- | --- | --- | --- | --- |
| STH epidemiology | Intensity of STH transmission | Prevalence of each STH species | |  |
|  | Environmental exposure | Access to sanitation and water  Socioeconomic conditions | |  |
| Capacity to deliver | Current implementation of STH control | National STH treatment coverage | |  |
|  | Health systems | Health expenditure | |  |
|  |  | Hospital beds | |  |
|  |  | Antenatal care | |  |
|  |  | Tuberculosis treatment completion | |  |
|  |  | Under five mortality rate | |  |
|  | Education systems | Education expenditure | |  |
|  |  | Primary school pupil: teacher ratios | |  |
|  |  | Primary school completion rate | |  |
|  |  | Youth literacy rates | |  |
|  | Delivery platforms | Vaccination coverage | |  |
|  |  | Net primary school enrolment | |  |
|  |  | Existence of, and national coverage rates for, LF programme | |  |
|  | Programme funding | External funding / partnership for NTD programmes | |  |
| Operational & financial feasibility | Logistics and infrastructure | Logistics performance | |  |
|  |  | Population within 4 hours travel time of city | |  |
|  | Governance | Political stability | |  |
|  |  | Control of corruption | |  |
|  |  | Fragility of state | |  |
|  | Economy | GDP per capita | |  |

**Table A. STH elimination feasibility framework- domains and indicators included in the feasibility analysis and components that make up each indicator as described in Brooker *et al.* [**[**1**](#_ENREF_1)**].**

| **Indicator** | **Component** | **Source** |
| --- | --- | --- |
| Environmental conditions | Land surface temperature (LST) (^o^C) (1km resolution) | Africa Soil Information System [[4](#_ENREF_4)] |
|  | Aridity index (AI) (1km resolution) | CGIAR-CSI [[5](#_ENREF_5)] |
|  | Enhanced vegetation index (EVI) (1km resolution) | Africa Soil Information System [[4](#_ENREF_4)] |
|  | Population density (Pop per 100 m^2^, average 2010-2015) (100m resolution) | WorldPop [[6](#_ENREF_6)] |
| Intensity of transmission | Baseline prevalence (%) | Baseline survey |
|  | Baseline average intensity of infection (epg) | Baseline survey |
| Location WASH and socioeconomic indicators | Socioeconomic PCA score (rescaled 0-100) | 2009 Population and Housing Census [[7](#_ENREF_7)] |
|  | Access improved sanitation (waterborne) (%) | 2009 Population and Housing Census [[7](#_ENREF_7)] |
|  | Access improved sanitation (waterborne, VIP& covered pit) (%) | 2009 Population and Housing Census [[7](#_ENREF_7)] |
|  | Access any sanitation (%) | 2009 Population and Housing Census [[7](#_ENREF_7)] |
|  | Access improved drinking water (%) | 2009 Population and Housing Census [[7](#_ENREF_7)] |
|  | Access piped drinking water (%) | 2009 Population and Housing Census [[7](#_ENREF_7)] |
|  | School attendance (%) | 2009 Population and Housing Census [[7](#_ENREF_7)] |
| School WASH | Availability of hand washing facility (yes/no) | Baseline survey |
|  | Type of water source | Baseline survey |
|  | Sanitation (VIP & waterborne/ pit latrine) | Baseline survey |
|  | Number of children per toilet | Baseline survey |
|  | Proportion of toilets clean (%) | Baseline survey |
|  | Any health programme (yes/no) | Baseline survey |
| Treatment | Year 1 treatment coverage (%) | Innovations for Poverty Action (IPA) |
|  | Year 2 treatment coverage (%) | IPA |
|  | Time since year 2 treatment (days) | IPA, Y3preMDA survey |

**Table B. School and location level components included in the analysis of factors associated with programme impact.**

| **Indicator** | **Component** | **Data source** | **PCA loading (weight**^1^**)** |
| --- | --- | --- | --- |
| Health systems | Qualified medical assistant during birth (%) | County Fact Sheets 2013 [[3](#_ENREF_3)] | 0.20 (0.04) |
|  | Births delivered at health care facility, 2009 (%) | Kenya CHFS 2013 [[2](#_ENREF_2)] | 0.23 (0.05) |
|  | Nurses, 2012 (per 100,000) | County Fact Sheets 2013 [[3](#_ENREF_3)] | 0.44 (0.20) |
|  | Doctors, 2012 (per 100,000) | County Fact Sheets 2013 [[3](#_ENREF_3)] | 0.35 (0.12) |
|  | Clinical officers, 2012 (per 100,000) | County Fact Sheets 2013 [[3](#_ENREF_3)] | 0.41 (0.17) |
|  | Preventive care budget, 2011 (KES per capita) | County Fact Sheets 2013 [[3](#_ENREF_3)] | 0.33 (0.11) |
|  | Curative care budget, 2011 (KES per capita) | County Fact Sheets 2013 [[3](#_ENREF_3)] | 0.38 (0.15) |
|  | National Hospital Insurance Fund coverage, 2012 (%) | County Fact Sheets 2013 [[3](#_ENREF_3)] | 0.33 (0.11) |
|  | Health spending, 2009 (per capita) | Kenya Open Data [[3](#_ENREF_3)] | 0.23 (0.05) |
| Education systems | Complete primary or secondary education, 2009 (%) | County Fact Sheets 2013 [[3](#_ENREF_3)] | 0.34 (0.12) |
|  | Literacy, 2009 (%) | County Fact Sheets 2013 [[3](#_ENREF_3)] | 0.72 (0.51) |
|  | Average pupil to teacher ratio, 2007 | Kenya Open Data [[3](#_ENREF_3)] | -0.61 (0.37) |
| Delivery platforms | Full immunisation coverage, 2012 (%) | Kenya CHFS 2013 [[2](#_ENREF_2)] | 0.71 (0.5) |
|  | Fully immunised under 1 year olds, 2012/2013 (%) | County Fact Sheets 2013 [[3](#_ENREF_3)] | 0.71 (0.5) |
| Logistics | Share of urban population, 2009 (%) | County Fact Sheets 2013 [[3](#_ENREF_3)] | 0.59 (0.34) |
|  | Paved roads, 2012 (%) | County Fact Sheets 2013 [[3](#_ENREF_3)] | 0.54 (0.30) |
|  | Households with electricity, 2009 (%) | County Fact Sheets 2013 [[3](#_ENREF_3)] | 0.60 (0.36) |
| Economy | Revenue, 2013/14 (per capita) | CRA County Budget 2014 [[8](#_ENREF_8)] | 0.71 (0.5) |
|  | Expenditures, 2013/14 (per capita) | CRA County Budget 2014 [[8](#_ENREF_8)] | 0.71 (0.5) |

^1^ normalised squared PCA loadings

**Table C.** **County indicators and components.** Variables were combined into indicators using PCA and the scores of the first component were used for further analysis.

**1.2. Correlation among covariates**

The correlation among investigated variables was assessed by pair wise correlation. Table D provides the pair wise correlation coefficients (r) for variables with r <= -0.4 (indicative of a negative correlation) or r ≥0.4 (indicative of positive correlation). Correlations were mainly observed among environmental variables and among socioeconomic and WASH access variables.

|  | LST | Aridity | EVI | Pop. | Socioec. | I. san | A. san. | I. drink | Hw | Clean t. | Edu. | Health | Logistics | Delivery | Econ. |
| --- | --- | --- | --- | --- | --- | --- | --- | --- | --- | --- | --- | --- | --- | --- | --- |
| LST | 1.00 |  |  |  |  |  |  |  |  |  |  |  |  |  |  |
| Aridity | -0.36 | 1.00 |  |  |  |  |  |  |  |  |  |  |  |  |  |
| EVI | -0.69 | 0.26 | 1.00 |  |  |  |  |  |  |  |  |  |  |  |  |
| Population | -0.29 | 0.48 | 0.17 | 1.00 |  |  |  |  |  |  |  |  |  |  |  |
| Socioeconomic score | 0.17 | 0.22 | -0.13 | 0.26 | 1.00 |  |  |  |  |  |  |  |  |  |  |
| Improved sanitation | 0.04 | 0.25 | -0.03 | 0.32 | 0.45 | 1.00 |  |  |  |  |  |  |  |  |  |
| Any sanitation | -0.01 | 0.26 | 0.00 | 0.38 | 0.48 | 0.64 | 1.00 |  |  |  |  |  |  |  |  |
| Improved drink water | 0.30 | 0.07 | -0.40 | 0.12 | 0.44 | 0.44 | 0.36 | 1.00 |  |  |  |  |  |  |  |
| Hw facility in school | 0.10 | 0.00 | -0.16 | -0.14 | -0.02 | 0.02 | -0.05 | 0.12 | 1.00 |  |  |  |  |  |  |
| Clean toilets | 0.26 | -0.05 | -0.39 | -0.10 | 0.15 | 0.06 | 0.04 | 0.16 | 0.41 | 1.00 |  |  |  |  |  |
| Education score | -0.46 | 0.14 | 0.53 | 0.20 | -0.21 | -0.04 | 0.11 | -0.46 | -0.20 | -0.25 | 1.00 |  |  |  |  |
| Health score | 0.07 | 0.00 | 0.07 | 0.22 | 0.30 | 0.07 | 0.15 | 0.16 | -0.01 | 0.06 | -0.05 | 1.00 |  |  |  |
| Logistics score | -0.06 | 0.18 | 0.17 | 0.40 | 0.28 | 0.16 | 0.22 | 0.11 | -0.06 | -0.05 | 0.27 | 0.48 | 1.00 |  |  |
| HS delivery score | -0.25 | 0.47 | 0.19 | 0.42 | 0.26 | 0.24 | 0.31 | 0.27 | -0.17 | -0.06 | 0.31 | 0.31 | 0.54 | 1.00 |  |
| Economy score | -0.06 | 0.26 | 0.05 | 0.25 | 0.16 | 0.39 | 0.09 | 0.19 | -0.11 | -0.09 | -0.32 | -0.05 | 0.01 | 0.14 | 1.00 |
| County | -0.21 | 0.24 | 0.28 | 0.17 | -0.09 | 0.02 | -0.18 | -0.28 | -0.07 | -0.11 | 0.03 | 0.05 | 0.43 | 0.04 | 0.55 |

(LST) land surface temperature, (EVI) enhanced vegetation index, (Pop.) population density, (Socioec.) socioeconomic indicator, (I.san) improved sanitation (waterborne, VIP or covered pit), (A.san) any sanitation, (I.drink) improved drinking water, (Hw) hand washing facility in school, (Clean t.) proportion of clean school toilets, (Edu.) county education index score, (Health) county health system index score, (Logistics) county logistics index score, (Delivery) county health service delivery index score, (Econ.) county economy index score

**Table D.** **Covariate pair wise correlation matrix.** Variables with any correlation of r<= -0.4 and =>0.4 (in grey) are included in the table.

**1.3. Geographical distribution of WASH access**

Fig A visualises the geographic distribution of community level access to improved sanitation (defined as waterborne, VIP or covered pit) in the study area and the type of sanitation facilities in surveyed schools. Community level access was highest for schools in locations in Kakamega, Vihigia and Kisumu Counties, while improved school sanitation facilities were found more frequently in Migori, Kisumu, Homa Bay and Bungoma.


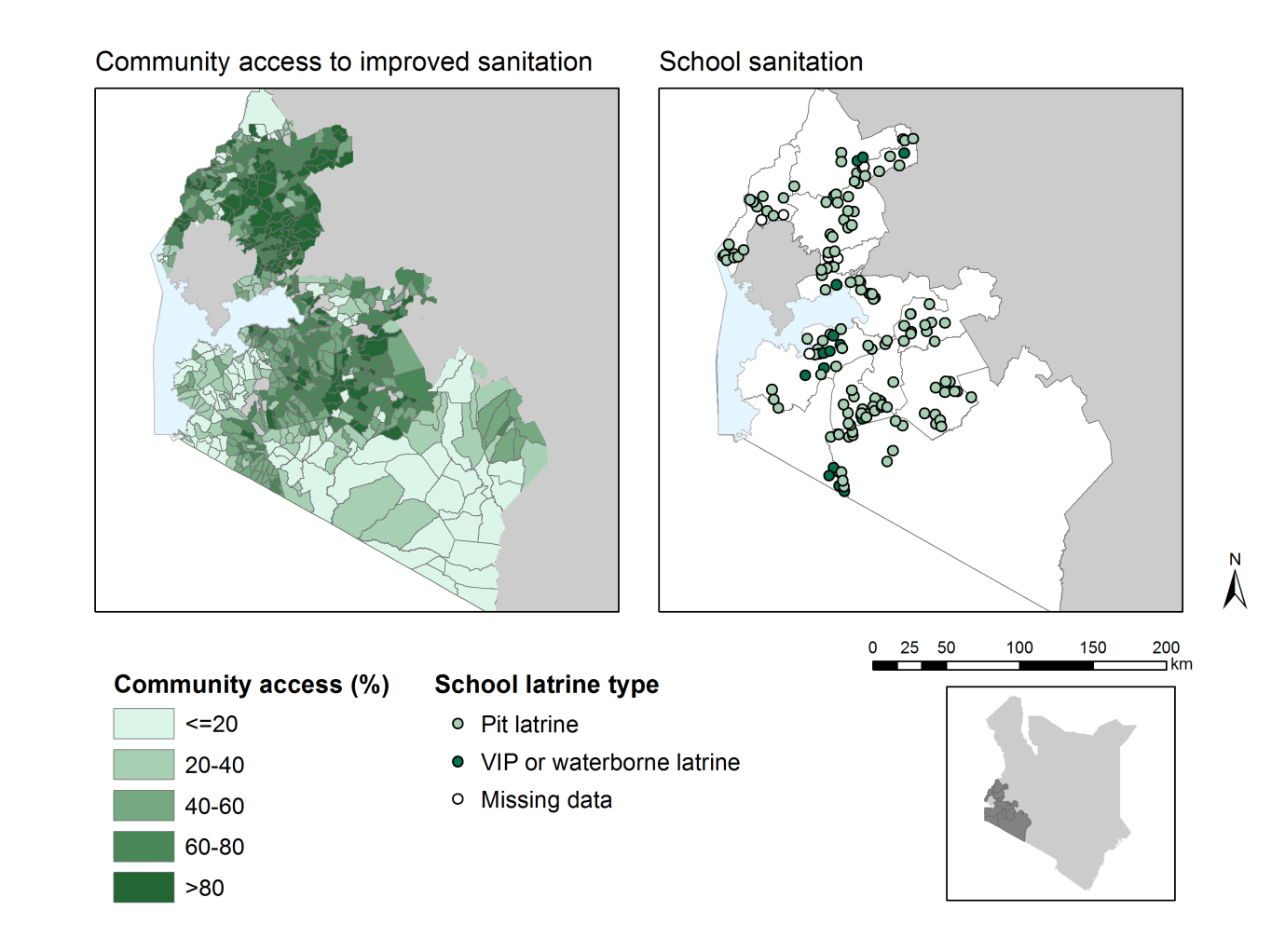


**Fig A. Geographic distribution of WASH indicators.** Left panel: community-level access to improved sanitation (waterborne, VIP or covered pit); right panel: school sanitation facility type.

**1.4. Geographical distribution of baseline and follow-up infection levels**

The geographical distribution of *A. lumbricoides* and hookworm baseline and follow-up infection levels (prevalence and average intensity of infection) together with relative reductions are visualised in Fig B and Fig C.

**
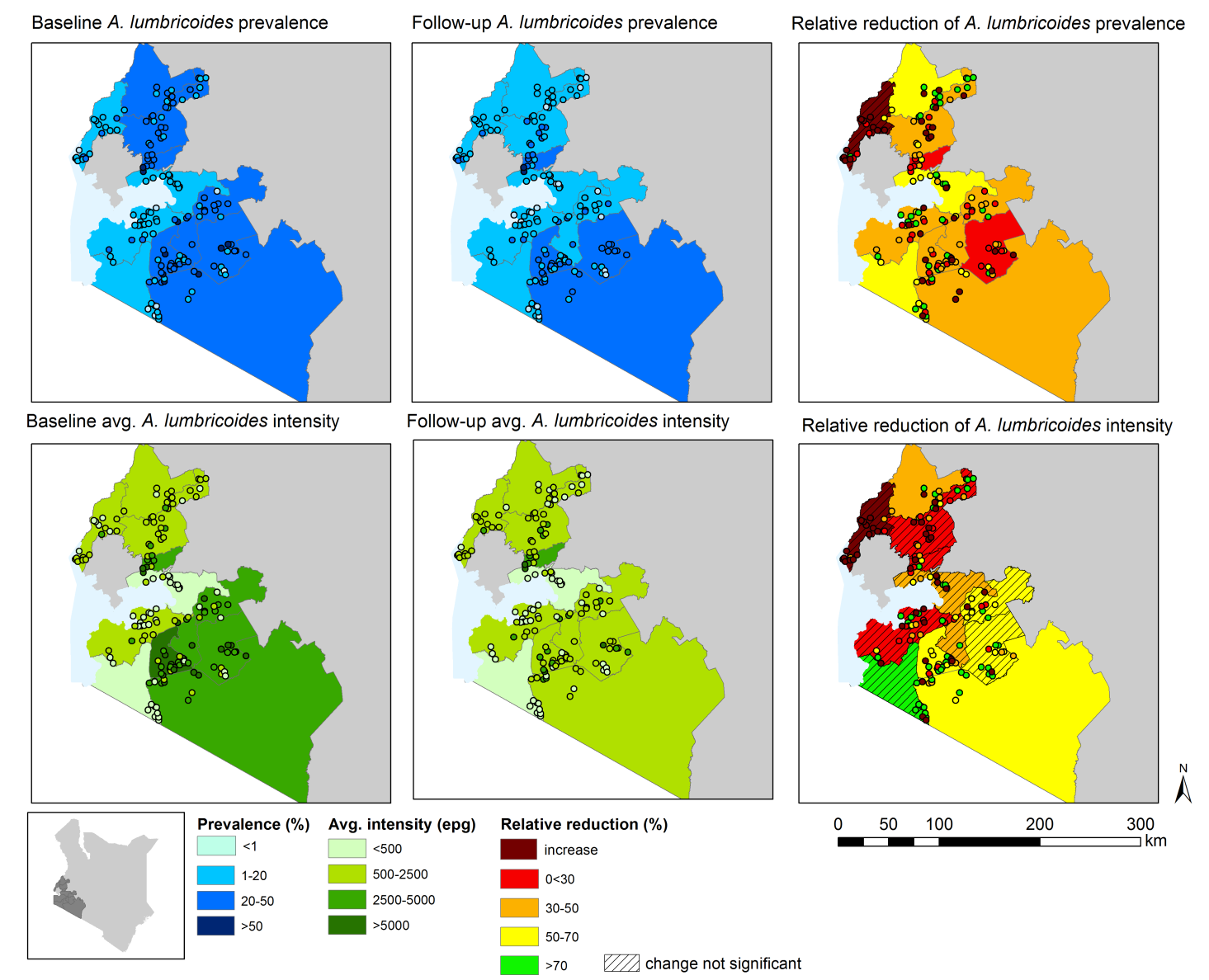
**

**Fig B. *A. lumbricoides* infections (prevalence and average intensity) at baseline (2012) and follow-up (2014) and relative reductions by school and county.**

**
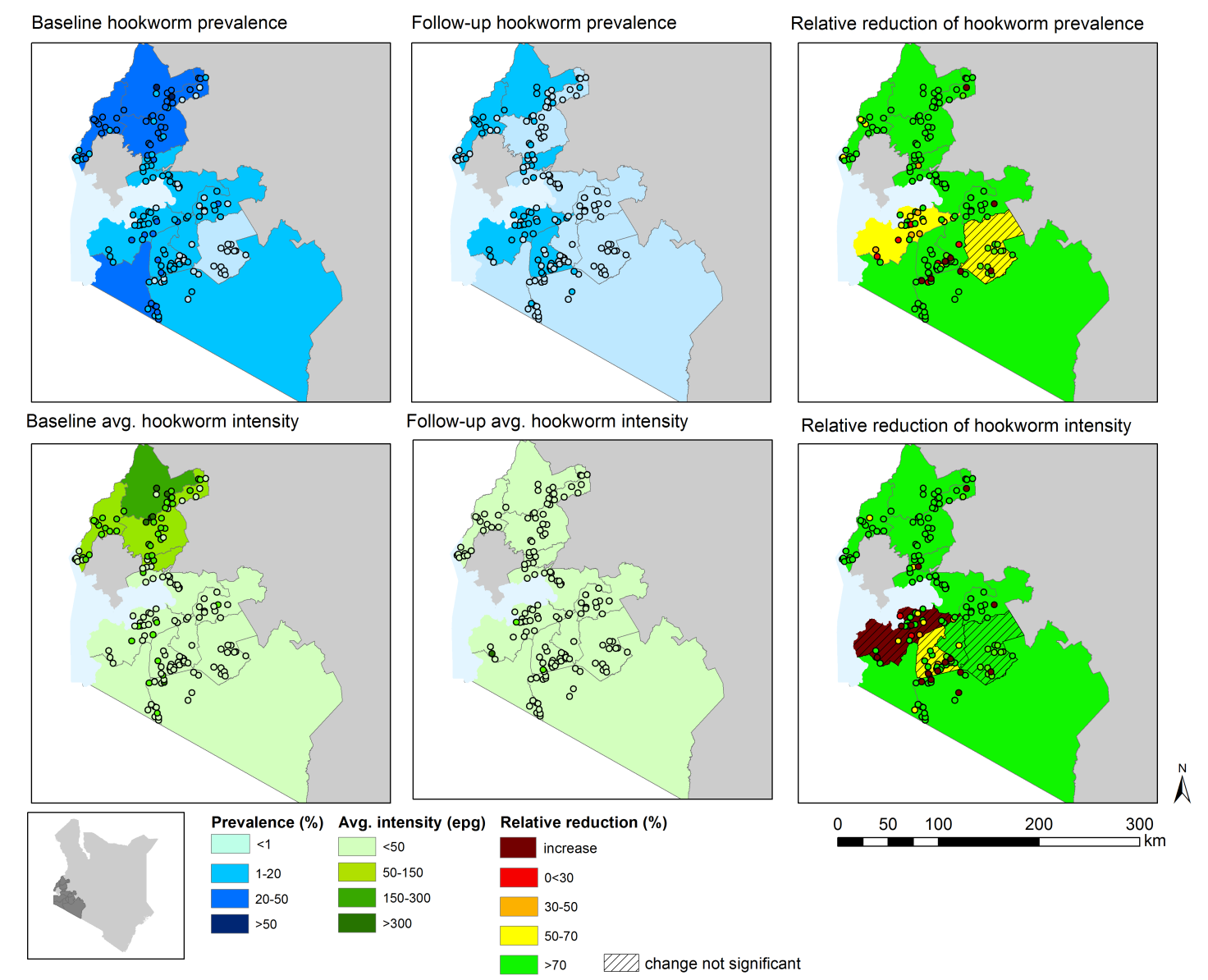
**

**Fig C. Hookworm infections (prevalence and average intensity) at baseline (2012) and follow-up (2014) and relative reductions by school and county.**

**1.5. Additional results- Univariable and multivariable analysis**

Detailed results of the univariable and multivariable analysis of factors associated with programme impact are summarised in Tables E and F.

|  |  | ***A. lumbricoides*** | | **Hookworm** | |
| --- | --- | --- | --- | --- | --- |
|  |  | **Prevalence reduction** | **Average epg**  **reduction** | **Prevalence reduction** | **Average epg**  **reduction** |
| **Variable** | **Categories** | **Coeff. (95%CI)^1^** | **Coeff. (95%CI) ^1^** | **Coeff. (95%CI) ^1^** | **Coeff. (95%CI) ^1^** |
| **LST** | <30^o^C | **base** | **base** | base | base |
|  | 30-35 ^o^C | **-4.10 (-7.19; -0.22)** | **-327.23 (-777.85; 245.78)** | 0.71 (0.08; 1.67) | 3.45 (-10.39; 18.14) |
|  | ≥35 ^o^C | **-8.27 (-11.84; -3.62)** | **-719.24 (-1241.44; -181.52)** | -0.70 (-2.95; 1.75) | -9.15 (-33.39; 3.42) |
| **Aridity Index** | <0.8 (more arid) | **base** | base | base | base |
|  | 0.8-1.0 | **3.5 (0.16; 6.95)** | 270.19 (-53.66; 615.61) | 0.74 (-0.46; 2.16) | -5.88 (-21.36; 3.35) |
|  | ≥1.0 (less arid) | **4.07 (-2.06; 9.34)** | -3.68 (-670.74; 606.89) | 0.06 (-1.01; 1.39) | -6.61 (-21.36; 2.59) |
| **EVI** | <0.4 | base | base | base | base |
|  | ≥0.4 | 2.73 (-0.89; 6.24) | -133.38 (-610.80; 239.79) | 0.11 (-0.69; 1.26) | -5.04 (-12.73; 1.85) |
| **Population density (per 100m^2^)** | <5 | **base** | **base** | base | **base** |
|  | 5-10 | **4.78 (1.28; 7.82)** | **568.43 (261.73; 958.45)** | -0.64 (-1.42; 0.04) | **-6.78 (-16.53; -1.28)** |
|  | ≥10 | **4.30 (-1.43; 9.12)** | **257.08 (-348.71; 910.97)** | -0.54 (-1.38; 0.32) | **0.38 (-11.27; 13.48)** |
| **Baseline prevalence** | <20% | NA | **base** | NA | base |
|  | 20-40% |  | **235.36 (-165.32; 618.64)** |  | 6.11 (-4.45; 17.92) |
|  | ≥40% |  | **604.63 (41.18; 1,305.76)** |  | 6.16 (-3.88; 22.41) |
| **Baseline intensity (*A. lumbricoides* / hookworm)** | <1500 epg / <100 epg | base | NA | base | NA |
|  | 1500-3000 epg/ 100-200 epg | 0.43 (-5.45; 6.53) |  | -1.02 (-2.92; 1.09) |  |
|  | >3000 epg / >200 epg | 5.99 (-1.19; 14.11) |  | -2.06 (-4.15; 0.03) |  |
| **Y1 treatment coverage** | <80% | base | base | base | base |
|  | 80-90% | 3.19 (-0.76; 8.41) | 238.85 (-326.76; 836.37) | -0.87 (-3.71; 1.51) | 5.70 (-20.27; 44.84) |
|  | ≥90% | 0.80 (-2.44; 5.30) | 27.65 (-523.11; 565.08) | -1.31 (-3.88; 0.37) | -8.46 (-29.52; 2.69) |
| **Y2 treatment coverage** | <90% | base | base | base | base |
|  | ≥90% | 2.15 (-1.77; 5.73) | 66.26 (-350.17; 413.66) | -0.15 (-1.38; 0.96) | 3.74 (-1.96; 13.77) |
| **Time since Y2 treatment** | <250 d | base | base | **base** | base |
|  | 250-300 d | -1.54 (-6.81; 3.19) | 11.90 (-565.14; 781.00) | **0.38 (-0.28; 1.03)** | 7.86 (-0.95; 18.93) |
|  | 300-350 d | -0.85 (-6.01; 3.83) | 10.78 (-561.48; 586.93) | **-0.47 (-1.17; 0.20)** | 4.01 (-1.92; 14.08) |
|  | ≥350 d | 0.27 (-5.59; 4.93) | 58.71 (-518.46; 855.08) | **2.05 (0.04; 4.06)** | 3.01 (-4.10; 10.48) |
| **Socioeconomic score** | <20 | base | base | base | **base** |
|  | ≥20 | -2.13 (-4.93; 0.57) | -236.63 (-579.40; 125.89) | -0.33 (-1.43; 0.64) | **-10.05 (-29.30; -1.05)** |
| **Access improved sanitation (waterborne)** | <5% | base | base | base | base |
|  | ≥5% | 0.92 (-2.58; 3.77) | -107.20 (-473.24; 251.68) | -0.40 (-1.35; 0.66) | -4.21 (-16.53; 2.72) |
| **Access improved sanitation (waterborne, VIP & covered pit)** | <50% | base | base | base | base |
|  | 50-75% | -1.27 (-4.48; 1.67) | -289.86 (-651.84; 69.64) | 0.21 (-1.00; 1.51) | -9.39 (-36.54; 3.22) |
|  | ≥75% | -0.97 (-4.87; 3.49) | -76.90 (-464.36; 411.20) | -1.10 (-2.36; 0.09) | -11.42 (-39.15; 1.17) |
| **Access any sanitation** | <50% | base | base | base | base |
|  | 50-75% | -2.22 (-7.83; 2.49) | -406.76 (-993.62; 75.25) | -1.14 (-4.54; 1.96) | -21.93 (-101.17; 15.53) |
|  | ≥75% | -1.44 (-6.38; 3.15) | -315.99 (-929.15; 191.73) | -1.50 (-4.14; 0.69) | -26.54 (-95.95; 2.75) |
| **Access improved drinking water** | <50% | base | base | base | base |
|  | 50-75% | 0.81 (-2.26; 4.56) | 135.69 (-344.50; 715.23) | 0.51 (-0.82; 1.93) | 0.98 (-11.25; 11.53) |
|  | ≥75% | -2.95 (-6.71; 0.15) | 65.05 (-261.78; 632.92) | -0.60 (-1.78; 0.54) | -4.97 (-12.37; 2.23) |
| **Access piped water** | <10% | base | base | base | **base** |
|  | ≥10% | -1.04 (-4.35; 2.55) | -190.18 (-629.99; 207.44) | 0.14 (-0.54; 1.02) | **-5.39 (-11.55; -0.39)** |
| **School attendance** | <95% | base | base | base | base |
|  | ≥95% | -0.58 (-3.99; 2.49) | -184.21 (-549.68; 161.23) | -0.03 (-1.09; 0.93) | -6.52 (-31.92; 3.74) |
| **Hand washing at school** | no | base | base | base | base |
|  | yes | -0.78 (-3.60; 2.03) | -38.24 (-265.67; 226.92) | 0.49 (-0.52; 1.44) | -2.36 (-16.24; 5.08) |
| **School water source** | piped | base | base | **base** | **base** |
|  | borehole/well | 3.91 (-1.61; 9.18) | 178.45 (-316.77; 665.11) | **1.08 (-0.12; 2.27)** | **0.45 (-9.68; 5.41)** |
|  | rain | 1.73 (-2.79; 5.91) | -124.83 (-646.67; 390.18) | **1.20 (0.35; 2.35)** | **13.72 (0.34; 39.88)** |
|  | river | 2.52 (-1.88; 7.35) | 179.66 (-358.60; 750.32) | **0.94 (-0.12; 2.02)** | **1.84 (-5.97; 7.46)** |
|  | others | -0.31 (-7.70; 6.14) | -302.57 (-1,091.14; 419.12) | **-1.57 (-2.08; 1.49)** | **-6.16 (-26.50; 0.26)** |
| **School sanitation** | pit latrine | **base** | base | base | base |
|  | VIP& waterborne | **-4.54 (-7.97; -1.73)** | -264.86 (-582.37; 20.55) | 1.30 (-1.10; 3.48) | -2.83 (-20.82; 3.99) |
| **Children per toilet** | <25 | base | base | base | **base** |
|  | 25-50 | -0.99 (-4.87; 2.26) | 142.72 (-257.64; 594.80) | 0.63 (-0.04; 1.59) | **3.60 (0.88; 6.92)** |
|  | ≥50 | 0.04 (-4.91; 4.20) | 270.35 (-210.80; 761.99) | -0.55 (-1.58; 0.61) | **3.23 (-3.22; 14.53)** |
| **Proportion toilets cleaned** | <25% | base | base | base | base |
|  | 25-50% | -0.98 (-4.94; 3.36) | -28.30 (-606.79; 504.23) | -0.14 (-0.70; 0.44) | 0.59 (-6.89; 10.04) |
|  | ≥50% | -3.32 (-7.61; 0.74) | -66.96 (-563.69; 412.99) | 0.54 (-0.16; 1.37) | -1.10 (-8.11; 3.32) |
| **Health programme** | no | base | base | base | base |
|  | yes | 0.20 (-2.90; 2.59) | 131.45 (-164.94; 456.94) | 0.83 (-0.02; 1.74) | -5.37 (-24.43; 4.75) |
| **County education score** | 1^st^ tertile | base | base | base | **base** |
|  | 2^nd^ tertile | -2.13 (-5.50; 1.18) | 86.60 (-309.41; 509.18) | 0.57 (-0.31; 1.58) | **13.87 (2.25; 35.68)** |
|  | 3^rd^ tertile | 0.29 (-2.92; 4.17) | -363.26 (-833.22; 101.86) | -0.12 (-0.71; 0.45) | **-1.94 (-7.32; 3.64)** |
| **County health system score** | 1^st^ tertile | base | **base** | **base** | base |
|  | 2^nd^ tertile | 3.14 (-0.57; 6.41) | **638.22 (189.14; 1,055.07)** | **1.49 (0.66; 2.33)** | -4.53 (-15.48; 4.43) |
|  | 3^rd^ tertile | 1.96 (-1.37; 5.12) | **326.64 (-16.57; 696.83)** | **0.89 (0.21; 1.60)** | -8.50 (-20.16; 0.93) |
| **County logistics score** | 1^st^ tertile | base | base | **base** | **base** |
|  | 2^nd^ tertile | -1.93 (-5.33; 1.48) | 88.40 (-221.09; 425.73) | **-0.98 (-1.99; 0.13)** | **11.36 (2.90; 25.04)** |
|  | 3^rd^ tertile | -0.22 (-3.42; 3.12) | 262.84 (-145.37; 765.08) | **-0.89 (-1.63; -0.29)** | **3.04 (0.01; 7.42)** |
| **County health service delivery score** | 1^st^ tertile | base | **base** | **base** | **base** |
|  | 2^nd^ tertile | 1.03 (-1.94; 3.97) | **371.92 (77.84; 663.79)** | **-1.29 (-2.19; -0.36)** | **-9.68 (-23.21; -0.29)** |
|  | 3^rd^ tertile | -0.67 (-4.50; 2.81) | **364.85 (-89.15; 899.86)** | **-1.52 (-2.51; -0.53)** | **-6.43(-19.24; 5.50)** |
| **County economy score** | 1^st^ tertile | base | base | **base** | base |
|  | 2^nd^ tertile | -2.38 (-5.51; 1.21) | 160.83 (-266.28; 547.94) | **-1.90 (-2.90; -0.96)** | -8.11 (-20.30; 0.44) |
|  | 3^rd^ tertile | -1.76 (-5.31; 1.38) | -147.75 (-512.75; 203.55) | **-1.52 (-2.24; -0.75)** | -8.17 (-30.34; 1.36) |

^1^Bias corrected 95% CI

**Table E. Univariable analysis of factors associated with programme impact measured as absolute change since baseline survey.** Estimates were obtained by univariable mixed effects linear regression analysis adjusting for baseline infections and with a random intercept for counties. Variables with coefficient CIs not overlapping zero were considered in the multivariable analysis (indicated in bold).

|  |  | ***A. lumbricoides*** | | **Hookworm** | |
| --- | --- | --- | --- | --- | --- |
|  |  | **Prevalence reduction^1^** | **Average epg**  **reduction** | **Prevalence reduction^2^** | **Average epg reduction^2^** |
| **Variable** | **Categories** | **Coefficient (95%CI)^3^** | **Coefficient (95%CI)^3^** | **Coefficient (95%CI)^3^** | **Coefficient (95%CI)^3^** |
| **LST** | <30^o^C | ***base*** | ***base*** | base | base |
|  | 30-35 ^o^C | ***-3.76 (-6.85; 0.58)*** | ***-201.04 (-623.51; 353.47)*** | 0.13 (-0.61; 0.85) | -3.93 (-20.30; 8.09) |
|  | ≥35 ^o^C | ***-7.60 (-11.55; -2.91)*** | ***-680.28 (-1,090.53; -144.63)*** | -1.65 (-3.93; 1.04) | -14.23 (-39.71; 2.66) |
| **Aridity Index** | <0.8 (more arid) | base | base | base | base |
|  | 0.8-1.0 | 2.44 (-0.82; 6.00) | 209.80 (-170.77; 543.04) | 0.74 (-0.43; 2.02) | -4.08 (-18.46; 3.17) |
|  | ≥1.0 (less arid) | 3.01 (-3.86; 9.09) | -161.72 (-806.72; 445.60) | 0.58 (-0.60; 2.17) | 4.25 (-1.85; 13.94) |
| **EVI** | <0.4 | base | **base** | base | base |
|  | ≥0.4 | -0.85 (-4.98; 2.70) | **-503.34 (-1,437.99; -75.24)** | 0.29 (-0.58; 1.53) | -0.40 (-12.16; 7.07) |
| **Population density (per 100m^2^)** | <5 | base | **base** | base | base |
|  | 5-10 | 3.61 (-0.01; 7.73) | **470.00 (188.08; 829.00)** | -0.63 (-1.37; 0.01) | -2.77 (-11.40; 0.58) |
|  | ≥10 | 2.97 (-3.64; 8.80) | **103.64 (-490.25; 903.07)** | 0.25 (-0.89; 1.51) | 10.63 (-0.99; 26.97) |
| **Baseline prevalence** | <20% | NA | **base** | NA | base |
|  | 20-40% |  | **137.90 (-224.24; 581.53)** |  | 7.05 (-5.50; 18.29) |
|  | ≥40% |  | **614.58 (51.51; 1,395.64)** |  | -7.64 (-43.83; 13.82) |
| **Baseline intensity (*A. lumbricoides* / hookworm)** | <1500 epg / <100 epg | base | NA | base | NA |
|  | 1500-3000 epg/ 100-200 epg | -1.14 (-8.10; 3.96) |  | -1.01 (-2.98; 0.96) |  |
|  | >3000 epg / >200 epg | 4.45 (-4.57; 12.48) |  | -1.56 (-3.57; 0.46) |  |
| **Y1 treatment coverage** | <80% | base | base | base | base |
|  | 80-90% | 5.06 (0.93; 12.00) | 243.07 (-264.56; 848.68) | 0.12 (-1.64; 2.00) | 7.06 (-21.60; 42.10) |
|  | ≥90% | 1.00 (-2.73; 6.50) | -37.50 (-550.33; 439.46) | -0.38 (-1.70; 0.92) | -4.82 (-26.87; 7.64) |
| **Y2 treatment coverage** | <90% | base | base | base | base |
|  | ≥90% | 1.08 (-3.13; 4.92) | 4.60 (-460.51; 332.47) | -0.32 (-1.56; 0.79) | 6.26 (-1.37; 20.05) |
| **Time since Y2 treatment** | <250 d | **base** | base | base | base |
|  | 250-300 d | **0.70 (-3.67; 5.06)** | 123.08 (-500.60; 895.54) | -0.13 (-1.01; 0.69) | 7.28 (-5.61; 25.72) |
|  | 300-350 d | **0.43 (-4.43; 4.61)** | 43.71 (-456.76; 609.73) | -0.64 (-1.54; 0.13) | 6.24 (-4.76; 18.76) |
|  | ≥350 d | **5.04 (0.38; 11.42)** | 226.02 (-412.39; 1,059.72) | 1.25 (-0.43; 3.21) | 1.61 (-15.74; 14.21) |
| **Socioeconomic score** | <20 | base | base | base | ***base*** |
|  | ≥20 | -1.92 (-4.58; 1.47) | -272.76 (-616.92; 139.43) | -0.61 (-1.86; 0.52) | ***-8.48 (-26.06; 0.00)*** |
| **Access improved sanitation (waterborne)** | <5% | base | base | base | base |
|  | ≥5% | 0.63 (-2.49; 3.80) | -152.98 (-501.60; 206.36) | -0.36 (-1.29; 0.56) | -2.89 (-20.23; 3.76) |
| **Access improved sanitation (waterborne, VIP & covered pit)** | <50% | base | **base** | **base** | base |
|  | 50-75% | -1.87 (-5.08; 0.53) | **-338.26 (-702.91; -43.68)** | **0.38 (-0.89; 1.48)** | -6.55 (-25.53; 4.29) |
|  | ≥75% | -0.22 (-4.28; 3.81) | **-127.92 (-531.20; 334.91)** | **-1.31 (-2.77; -0.12)** | -4.17 (-21.85; 2.56) |
| **Access any sanitation** | <50% | base | base | base | base |
|  | 50-75% | -1.21 (-6.89; 3.46) | -421.30 (-1047.64; 68.17) | -0.05 (-2.57; 2.04) | -23.02 (-108.06; 20.24) |
|  | ≥75% | -0.89 (-5.82; 3.38) | -316.46 (-894.73; 212.74) | -0.74 (-2.63; 0.90) | -21.94 (-94.95; 12.40) |
| **Access improved drinking water** | <50% | base | base | base | **base** |
|  | 50-75% | 2.44 (-0.37; 7.45) | 232.48 (-171.64; 748.06) | 0.55 (-1.02; 1.87) | **9.78 (0.97; 23.58)** |
|  | ≥75% | -1.30 (-5.21; 2.37) | 245.86 (-28.95; 936.26) | -0.51 (-1.70; 0.66) | **2.03 (-5.62; 11.12)** |
| **Access piped water** | <10% | base | base | base | base |
|  | ≥10% | -1.62 (-4.78; 2.05) | -174.50 (-598.33; 227.75) | 0.30 (-0.64; 1.29) | -1.83 (-16.34; 2.28) |
| **School attendance** | <95% | base | base | base | base |
|  | ≥95% | -0.78 (-4.00; 2.78) | -202.87 (-558.69; 163.37) | 1.90 (-0.61; 1.14) | -4.11 (-25.14; 7.60) |
| **Hand washing at school** | no | base | base | base | base |
|  | yes | -0.79 (-3.57; 2.47) | -67.78 (-341.97; 201.75) | 0.15 (-0.79; 1.02) | -4.36 (-21.66; 4.17) |
| **School water source** | piped | base | base | ***base*** | ***base*** |
|  | borehole/well | 5.54 (-0.09; 10.75) | 231.02 (-239.11; 731.45) | ***1.07 (-0.18; 2.55)*** | ***1.95 (-5.04; 8.56)*** |
|  | rain | 1.50 (-3.48; 5.80) | -100.78 (-633.82; 329.13) | ***1.05 (0.18; 2.23)*** | ***13.71 (0.35; 39.99)*** |
|  | river | 3.08 (-1.76; 7.65) | 138.20 (-425.96; 686.50) | ***0.97 (0.03; 2.00)*** | ***1.94 (-5.52; 7.95)*** |
|  | others | -1.88 (-8.47; 4.42) | -486.92 (1,302.04; 97.47) | ***0.11 (-2.03; 2.13)*** | ***-3.32 (-15.77; 8.68)*** |
| **School sanitation** | pit latrine | ***base*** | base | base | base |
|  | VIP& waterborne | ***-3.53 (-7.41; -0.54)*** | 172.71 (-496.50; 141.08) | 0.57 (-1.21; 2.31) | -9.17 (-31.50; 2.17) |
| **Children per toilet** | <25 | base | base | base | **base** |
|  | 25-50 | -0.26 (-3.31; 3.68) | 195.38 (-180.85; 669.40) | 0.57 (-0.29; 1.53) | **3.89 (0.42; 8.84)** |
|  | ≥50 | 0.20 (-4.21; 4.35) | 324.07 (-168.11; 839.34) | -0.47 (-1.82; 0.89) | **2.48 (-4.87; 13.62)** |
| **Proportion toilets cleaned** | <25% | base | base | base | base |
|  | 25-50% | -1.74 (-5.84; 2.87) | -124.81 (-651.69; 375.82) | -0.12 (-0.94; 0.58) | -0.23 (-8.33; 9.22) |
|  | ≥50% | -1.97 (-5.81; 2.29) | -157.39 (-728.36; 309.65) | 0.37 (-0.45; 1.12) | -1.83 (-10.73; 2.62) |
| **Health programme** | no | base | base | base | base |
|  | yes | 1.03 (-1.73; 3.63) | 149.18 (-125.49; 452.18) | 0.72 (-0.07; 1.65) | -4.09 (-20.75; 7.61) |
| **County education score** | 1^st^ tertile | base | **base** | base | ***base*** |
|  | 2^nd^ tertile | -0.19 (-4.23; 3.22) | **219.50 (-281.22; 692.84)** | 1.45 (-0.31; 3.32) | ***12.73 (2.74; 32.00)*** |
|  | 3^rd^ tertile | -2.87 (-6.64; 0.81) | **-676.58 (-1,129.10; -246.78)** | 0.16 (-1.14; 1.75) | ***-3.95 (-17.51; 1.76)*** |
| **County health system score** | 1^st^ tertile | base | **base** | ***base*** | **base** |
|  | 2^nd^ tertile | 3.66 (-1.04; 6.85) | **711.08 (294.72; 1175.92)** | ***1.85 (0.79; 2.79)*** | **23.71 (4.89; 58.48)** |
|  | 3^rd^ tertile | 1.83 (-1.56; 4.98) | **387.42 (77.20; 750.22)** | ***1.17 (0.09; 2.13)*** | **22.94 (5.66; 58.45)** |
| **County logistics score** | 1^st^ tertile | base | base | base | base |
|  | 2^nd^ tertile | -1.94 (-5.08; 1.97) | 345.89 (-82.12; 779.11) | 2.34 (1.41; 3.55) | -1.76 (-13.24; 4.61) |
|  | 3^rd^ tertile | -0.02 (-3.80; 3.67) | 208.32 (-352.82; 777.27) | omitted | -2.52 (-16.04; 1.81) |
| **County health service delivery score** | 1^st^ tertile | base | base | ***base*** | base |
|  | 2^nd^ tertile | -0.10 (-3.31; 3.27) | 108.19 (168.70; 453.66) | ***-1.63 (-2.87; -0.39)*** | -0.42 (-6.42; 6.11) |
|  | 3^rd^ tertile | -1.53 (-6.23; 2.87) | 235.99 (-238.77; 722.44) | ***-1.33 (-2.63; -0.27)*** | 0.91 (-11.65; 8.48) |
| **County economy score** | 1^st^ tertile | base | base | ***base*** | **base** |
|  | 2^nd^ tertile | -1.78 (-5.35; 1.70) | 209.57 (-302.06; 677.31) | ***-0.68 (-1.55; 0.22)*** | **-13.39 (-37.08; -1.66)** |
|  | 3^rd^ tertile | -1.49 (-4.61; 1.62) | -126.97 (-501.71; 227.90) | ***-1.23 (-1.97; -0.49)*** | **-0.46 (-11.72; 8.89)** |
| **Random effect (sd)** | County | 2.23 (0.00; 3.75) | 274.23 (0.00; 541.04) | 0.53 (0.00; 0.88) | 0.00 (0.00; 4.95) |

^1^6 schools with missing school sanitation information were excluded from final models

^2^5 schools with missing school water source information were excluded from final models

^3^Bias corrected 95% CI

**Table F. Factors associated with programme impact measured as absolute change since baseline survey.** A negative coefficient indicates a greater absolute reduction. Estimates were obtained by multivariable mixed effects linear regression analysis adjusting for baseline infection and with a random intercept for counties. Final models were additionally adjusted for variables indicated in italic; variables with CIs not overlapping zero are indicated in bold.

**1.6. Missing data sensitivity analysis**

To investigate the sensitivity of final results to missing data, missing values were imputed as i) lowest and ii) highest values. Results are summarised in Tables G and H, respectively. Variables with 95% CIs close to zero (e.g. school water source, school health programmes) were most sensitive to the imputation of both low and high values, probably due to the inclusion of schools that were otherwise excluded from final models. Other variables (e.g. school sanitation) were affected only by imputation of lower or higher values. The imputation also affected a small number of associations of variables without missing data.

|  |  | ***A. lumbricoides*** | | **Hookworm** | |
| --- | --- | --- | --- | --- | --- |
|  |  | **Prevalence reduction** | **Average epg**  **reduction** | **Prevalence reduction** | **Average epg reduction** |
| **Variable** | **Categories** | **Coefficient (95%CI)^1^** | **Coefficient (95%CI)^1^** | **Coefficient (95%CI)^1^** | **Coefficient (95%CI)^1^** |
| **Variables with missing observations** | | | | | |
| **School water source** | piped | base | base | base | base |
|  | borehole/well | 3.62 (-1.69; 8.80) | -11.81 (-528.10; 504.75) | 0.11 (-1.95; 2.03) | 2.24 (-2.77; 8.46) |
|  | rain | 0.19 (-4.75; 4.12) | -331.16 (-985.64; 129.16) | 0.31 (-1.45; 1.60) | 14.07 (-0.82; 42.22) |
|  | river | 1.39 (-3.29; 6.14) | -110.59 (-698.93; 417.59) | 0.25 (-1.55; 1.63) | 2.41 (-2.91; 9.67) |
|  | others | -3.19 (-10.20; 3.51) | -755.91 (-1465.90; -110.94) | -0.68 (-3.77; 1.63) | -2.85 (-14.54; 11.64) |
| **Hand washing at school** | no | base | base | base | base |
|  | yes | -1.18 (-4.10; 1.40) | -88.32 (-351.09; 180.13) | 0.34 (-0.66; 1.37) | -3.98 (-19.17; 4.95) |
| **School sanitation** | pit latrine | base | base | base | base |
|  | VIP& waterborne | -2.60 (-5.73; 0.34) | -25.78 (-337.50; 353.85) | 0.85 (-0.79; 2.68) | 3.12 (-21.09; 12.62) |
| **Children per toilet** | <25 | base | base | base | base |
|  | 25-50 | -1.46 (-4.82; 1.87) | 2.31 (-439.54; 413.63) | 0.40 (-0.48; 1.42) | -8.41 (-42.25; 6.83) |
|  | ≥50 | -0.74 (-5.03; 3.46) | 148.83 (-341.39; 647.65) | -0.73 (-2.13; 0.52) | -10.99 (-45.23; 9.53) |
| **Proportion toilets cleaned** | <25% | base | base | base | base |
|  | 25-50% | -1.66 (-5.98; 2.18) | -143.11 (-647.12; 298.41) | -0.45 (-1.31; 0.30) | -11.23 (-40.12; 5.83) |
|  | ≥50% | -2.81 (-6.56; 0.68) | -235.55 (-707.79; 190.97) | 0.17 (-0.84; 1.18) | -11.44 (-42.53; 2.46) |
| **Health programme** | no | base | base | base | base |
|  | yes | 1.02 (-1.79; 3.83) | 149.18 (-150.00; 450.34) | 0.88 (0.06; 1.82) | -4.05 (-21.60; 8.02) |
| **Other variables with changed associations** | | | | | |
| **Population density (per 100m^2^)** | <5 | base | NC | NC | NC |
|  | 5-10 | 3.83 (0.28; 7.08) |  |  |  |
|  | ≥10 | 3.06 (-2.44; 8.79) |  |  |  |
| **Time since Y2 treatment** | <250 d | base | NC | NC | NC |
|  | 250-300 d | 1.11 (3.78; 5.73) |  |  |  |
|  | 300-350 d | 0.70 (-3.81; 4.90) |  |  |  |
|  | ≥350 d | 4.52 (-0.76; 9.39) |  |  |  |
| **Socioeconomic score** | <20 | NC | NC | NC | base |
|  | ≥20 |  |  |  | -4.39 (-18.59; 2.04) |
| **Access improved drinking water** | <50% | NC | NC | NC | base |
|  | 50-75% |  |  |  | 9.24 (-0.22; 21.48) |
|  | ≥75% |  |  |  | 1.05 (-6.71; 10.54) |
| **County education score** | 1^st^ tertile | NC | NC | base | NC |
|  | 2^nd^ tertile |  |  | 2.22 (0.25; 4.69) |  |
|  | 3^rd^ tertile |  |  | 0.69 (-0.90; 2.55) |  |
| **County health system score** | 1^st^ tertile | base | NC | NC | NC |
|  | 2^nd^ tertile | 3.97 (0.27; 7.27) |  |  |  |
|  | 3^rd^ tertile | 2.31 (-1.13; 5.43) |  |  |  |

^1^ Bias corrected confidence interval

NC- association not changed

**Table G. Results of the sensitivity analysis with low imputed values.** Associations that changed (95% CI previously not including zero now including zero or vice versa) compared to the main analysis are highlighted in grey.

|  |  | ***A. lumbricoides*** | | **Hookworm** | |
| --- | --- | --- | --- | --- | --- |
|  |  | **Prevalence reduction** | **Average epg**  **reduction** | **Prevalence reduction** | **Average epg reduction** |
| **Variable** | **Categories** | **Coefficient (95%CI)^1^** | **Coefficient (95%CI)^1^** | **Coefficient (95%CI)^1^** | **Coefficient (95%CI)^1^** |
| **Variables with missing observations** | | | | | |
| **School water source** | piped | base | base | base | base |
|  | borehole/well | 5.82 (0.17; 11.41) | 208.55 (-318.19; 731.18) | 0.84 (-0.48; 2.20) | 2.00 (-3.98; 7.67) |
|  | rain | 1.90 (-2.53; 6.68) | -97.62 (-716.50; 354.93) | 0.98 (-0.03; 1.96) | 13.73 (-0.05; 40.02) |
|  | river | 3.97 (-1.17; 8.83) | 227.17 (-334.38; 787.87) | 1.23 (0.21; 2.49) | 1.70 (-5.25; 7.90) |
|  | others | -1.32 (-8.44; 4.63) | -490.97 (-1270.57; 91.65) | 0.11 (-2.14; 2.52) | -3.24 (-18.10; 9.44) |
| **Hand washing at school** | no | base | base | base | base |
|  | yes | -0.73 (-3.39; 1.94) | -47.01 (-320.35; 212.11) | 0.31 (-0.75; 1.29) | -4.27 (-21.59; 4.29) |
| **School sanitation** | pit latrine | base | base | base | base |
|  | VIP& waterborne | -3.29 (-6.51; -0.23) | -179.57 (-441.15; 127.71) | 1.37 (-0.72; 3.77) | -9.78 (-31.09; 1.10) |
| **Children per toilet** | <25 | base | base | base | base |
|  | 25-50 | -0.67 (-4.19; 2.57) | 143.44 (-277.00; 630.32) | 0.72 (-0.20; 1.77) | 6.03 (1.24; 19.35) |
|  | ≥50 | 0.43 (-3.42; 4.83) | 354.74 (-108.35; 938.26) | -0.24 (-1.56; 1.03) | 15.79 (-3.87; 49.99) |
| **Proportion toilets cleaned** | <25% | base | base | base | base |
|  | 25-50% | -1.16 (-5.29; 2.91) | -35.13 (-654.59; 480.10) | 0.00 (-0.86; 0.79) | 6.87 (-5.18; 27.15) |
|  | ≥50% | -2.56 (-7.09; 1.77) | -192.38 (-772.57; 263.64) | 0.43 (-0.42; 1.35) | -3.03 (-16.60; 3.65) |
| **Health programme** | no | base | base | base | base |
|  | yes | 0.94 (-1.75; 3.75) | 149.18 (-126.28; 501.24) | 0.87 (0.08; 1.82) | -4.10 (-20.44; 8.12) |
| **Other variables with changed associations** | | | | | |
| **Population density (per 100m^2^)** | <5 | base | NC | NC | NC |
|  | 5-10 | 3.64 (0.13; 7.14) |  |  |  |
|  | ≥10 | 2.73 (-3.35; 8.07) |  |  |  |
| **Y1 treatment coverage** | <80% | base | NC | NC | NC |
|  | 80-90% | 4.16 (0.29; 9.72) |  |  |  |
|  | ≥90% | 0.96 (-2.71; 4.96) |  |  |  |
| **Socioeconomic score** | <20 | NC | NC | NC | NC |
|  | ≥20 |  |  |  | -8.21 (-25.31; 0.35) |
| **Access improved drinking water** | <50% | NC | NC | NC | base |
|  | 50-75% |  |  |  | 8.99 (-0.61; 20.41) |
|  | ≥75% |  |  |  | 0.99 (-8.42; 9.53) |
| **Baseline intensity (*A. lumbricoides* / hookworm)** | <1500 epg / <100 epg | NC | NA | base | NA |
|  | 1500-3000 epg/ 100-200 epg |  |  | -1.03 (-3.09; 0.93) |  |
|  | >3000 epg / >200 epg |  |  | -2.11 (-4.17; -0.01) |  |
| **County education score** | 1^st^ tertile | NC | NC | base | NC |
|  | 2^nd^ tertile |  |  | 1.86 (0.09; 4.11) |  |
|  | 3^rd^ tertile |  |  | 0.36 (-0.99; 1.82) |  |
| **County logistics score** | 1^st^ tertile | NC | NC | base | base |
|  | 2^nd^ tertile |  |  |  | -1.50 (-12.24; 4.88) |
|  | 3^rd^ tertile |  |  |  | -3.47 (-14.91; -0.08) |

^1^ Bias corrected confidence interval

NC- association not changed; NA- not applicable

**Table H. Results of the sensitivity analysis with high imputed values.** Associations that changed (95% CI previously not including zero now including zero or vice versa) compared to the main analysis are highlighted in grey.

**2. Factors associated with baseline infections**

To explore whether factors associated with programme impact were different from general risk factors of infection, we investigated associations of variables with *A. lumbricoides* and hookworm baseline infection levels (prevalence and intensity) using school level mixed effects logistic and negative binomial regression analysis, respectively, including a random intercept for counties. Univariable analysis was first carried out and variables with p-values <0.05 (based on likelihood ratio test) were considered for multivariable analysis. Final models were developed using a backward selection procedure, where variables were removed based on highest p-values and only variables with p<0.05 were retained. Results of the multivariable analysis are summarised in Table I and show that associations with baseline infections were partially different from those with programme impact.

|  |  | ***A. lumbricoides*** | | **Hookworm** | |
| --- | --- | --- | --- | --- | --- |
|  |  | **Prevalence** | **Intensity** | **Prevalence** | **Intensity** |
| **Variable** | **Categories** | **OR (95%CI)** | **Egg count ratio (95%CI)** | **OR (95%CI)** | **Egg count ratio (95%CI)** |
| **LST** | <30^o^C | ***base*** | ***base*** | ***base*** | base |
|  | 30-35 ^o^C | ***1.10 (0.93; 1.30)*** | ***0.69 (0.35; 1.36)*** | ***1.25 (0.98; 1.60)*** | 0.49 (0.17; 1.41) |
|  | ≥35 ^o^C | ***0.56 (0.41; 0.77)*** | ***0.23 (0.08; 0.68)*** | ***0.66 (0.46; 0.93)*** | 0.40 (0.10; 1.52) |
| **Aridity Index** | <0.8 (more arid) | ***base*** | ***base*** | ***base*** | base |
|  | 0.8-1.0 | ***1.57 (1.35; 1.81)*** | ***1.91 (1.16; 3.14)*** | ***1.17 (1.02; 1.35)*** | 1.45 (0.78; 2.69) |
|  | ≥1.0 (less arid) | ***1.28 (1.05; 1.55)*** | ***1.97 (0.96; 4.06)*** | ***1.60 (1.29; 1.99)*** | 1.07 (0.46; 2.48) |
| **EVI** | <0.4 | base | base | base | base |
|  | ≥0.4 | 1.06 (0.88; 1.27) | 1.87 (0.83; 4.19) | 0.98 (0.77; 1.25) | 1.75 (0.71; 4.28) |
| **Population density (per 100m^2^)** | <5 | ***base*** | base | ***base*** | base |
|  | 5-10 | ***1.25 (1.11; 1.42)*** | 1.41 (0.87; 2.29) | ***0.96 (0.84; 1.10)*** | 1.33 (0.76; 2.35) |
|  | ≥10 | ***0.90 (0.74; 1.10)*** | 1.16 (0.52; 2.58) | ***0.67 (0.52; 0.85)*** | 0.77 (0.32; 1.85) |
| **Socioeconomic score** | <20 | base | base | ***base*** | base |
|  | ≥20 | 0.93 (0.80; 1.07) | 0.94 (0.58; 1.52) | ***0.77 (0.65; 0.90)*** | 0.89 (0.48; 1.63) |
| **Access improved sanitation (waterborne)** | <5% | base | base | base | base |
|  | ≥5% | 1.00 (0.89; 1.13) | 0.95 (0.59; 1.54) | 0.91 (0.79; 1.05) | 0.72 (0.39; 1.32) |
| **Access impr. sanitation (waterborne, VIP & covered pit)** | <50% | ***base*** | base | base | base |
|  | 50-75% | ***1.11 (0.94; 1.32)*** | 1.07 (0.64; 1.81) | 1.03 (0.88; 1.20) | 1.18 (0.64; 2.19) |
|  | ≥75% | ***1.48 (1.20; 1.81)*** | 0.96 (0.48; 1.91) | 1.21 (0.98; 1.49) | 1.36 (0.58; 3.21) |
| **Access any sanitation** | <50% | ***base*** | base | **base** | base |
|  | 50-75% | ***0.73 (0.58; 0.93)*** | 0.35 (0.13; 0.90) | **0.75 (0.56; 1.00)** | 0.91 (0.25; 3.24) |
|  | ≥75% | ***0.97 (0.74; 1.27)*** | 0.76 (0.38; 1.50) | **0.64 (0.46; 0.88)** | 0.85 (0.26; 2.80) |
| **Access improved drinking water** | <50% | ***base*** | base | base | base |
|  | 50-75% | ***1.20 (1.03; 1.41)*** | 0.95 (0.54; 1.66) | 1.01 (0.84; 1.20) | 1.39 (0.71; 2.72) |
|  | ≥75% | ***0.98 (0.80; 1.19)*** | 0.83 (0.44; 1.58) | 1.08 (0.88; 1.33) | 1.20 (0.53; 2.71) |
| **Access piped water** | <10% | ***base*** | base | base | base |
|  | ≥10% | ***0.67 (0.59; 0.75)*** | 0.87 (0.52; 1.46) | 0.92 (0.79; 1.09) | 0.63 (0.35; 1.17) |
| **School attendance** | <95% | base | base | ***base*** | ***base*** |
|  | ≥95% | 0.90 (0.80; 1.02) | 1.03 (0.69; 1.54) | ***0.81 (0.72; 0.92)*** | ***0.49 (0.30; 0.81)*** |
| **Hand washing at school** | no | ***base*** | **base** | base | base |
|  | yes | ***0.82 (0.73; 0.92)*** | **0.55 (0.37; 0.81)** | 0.94 (0.85; 1.05) | 0.72 (0.44; 1.20) |
| **School water source** | piped | ***base*** | base | ***base*** | base |
|  | borehole/well | ***0.65 (0.54; 0.77)*** | 0.64 (0.33; 1.25) | ***0.98 (0.82; 1.17)*** | 0.69 (0.29; 1.65) |
|  | rain | ***0.99 (0.84; 1.16)*** | 1.21 (0.60; 2.44) | ***0.74 (0.62; 0.90)*** | 0.42 (0.17; 0,99) |
|  | river | ***0.96 (0.82; 1.12)*** | 1.24 (0.64; 2.40) | ***0.85 (0.71; 1.02)*** | 0.56 (0.25; 1.28) |
|  | others | ***0.83 (0.66; 1.05)*** | 1.14 (0.42; 3.07) | ***0.40 (0.29; 0.55)*** | 0.25 (0.07; 0.91 |
| **School sanitation** | pit latrine | ***base*** | base | **base** | base |
|  | VIP& waterborne | ***0.68 (0.55; 0.83)*** | 0.51 (0.25; 1.01) | **0.81 (0.69; 0.96)** | 0.77 (0.35; 1.69) |
| **Children per toilet** | <25 | ***base*** | base | ***base*** | base |
|  | 25-50 | ***1.03 (0.90; 1.17)*** | 0.98 (0.55; 1.75) | ***1.24 (1.02; 1.50)*** | 1.70 (0.85; 3.40) |
|  | ≥50 | ***1.40 (1.19; 1.64)*** | 0.77 (0.39; 1.52) | ***1.13 (0.91; 1.40)*** | 2.09 (0.94; 4.63) |
| **Proportion toilets cleaned** | <25% | ***base*** | **base** | **base** | base |
|  | 25-50% | ***0.68 (0.60; 0.78)*** | **0.61 (0.34; 1.10)** | **0.79 (0.65; 0.96)** | 0.79 (0.32; 1.94) |
|  | ≥25% | ***0.74 (0.64; 0.85)*** | **0.41 (0.25; 0.69)** | **0.73 (0.62; 0.84)** | 0.73 (0.36; 1.48) |
| **Health programme** | no | ***base*** | base | **base** | base |
|  | yes | ***0.80 (0.72; 0.89)*** | 0.71 (0.46; 1.12) | **0.77 (0.67;0.88)** | 0.82 (0.46; 1.46) |
| **County education score** | 1^st^ tertile | base | base | ***base*** | ***base*** |
|  | 2^nd^ tertile | 0.97 (0.44; 2.15) | 0.70 (0.31; 1.57) | ***1.79 (0.45; 7.07)*** | ***1.38 (0.17; 11.46)*** |
|  | 3^rd^ tertile | 1.56 (0.70; 3.48) | 2.10 (0.93; 4.75) | ***0.21 (0.05; 0.88)*** | ***0.05 (0.01; 0.39)*** |
| **County health system score** | 1^st^ tertile | base | base | **base** | base |
|  | 2^nd^ tertile | 1.57 (0.70; 3.54) | 1.28 (0.48; 3.46) | **1.84 (0.61; 5.54)** | 3.00 (0.49; 18.42) |
|  | 3^rd^ tertile | 1.23 (0.55; 2.78) | 1.47 (0.54; 3.97) | **5.78 (1.64; 20.41)** | 10.65 (1.40; 81.00) |
| **County logistics score** | 1^st^ tertile | base | base | base | base |
|  | 2^nd^ tertile | 0.63 (0.28; 1.41) | 0.57 (0.25; 1.26) | 4.68 (1.50; 14.61) | 6.69 (0.98; 45.72) |
|  | 3^rd^ tertile | 0.95 (0.43; 2.12) | 1.29 (0.61; 2.70) | 2.29 (0.71; 7.33) | 3.51 (0.50; 24.59) |
| **County health service delivery score** | 1^st^ tertile | base | **base** | **base** | **base** |
|  | 2^nd^ tertile | 0.56 (0.28; 1.11) | **0.53 (0.30; 0.94)** | **2.71 (0.98; 7.45)** | **3.77 (1.34; 10.62)** |
|  | 3^rd^ tertile | 1.28 (0.65; 2.54) | **1.22 (0.71; 2.08)** | **6.73 (2.12; 21.35)** | **38.08 (11.71; 123.76)** |
| **County economy score** | 1^st^ tertile | base | base | base | base |
|  | 2^nd^ tertile | 0.76 (0.33; 1.77) | 0.66 (0.26; 1.69) | 1.29 (0.27; 6.11) | 1.56 (0.14; 17.18) |
|  | 3^rd^ tertile | 0.94 (0.41; 2.15) | 1.19 (0.48; 2.92) | 2.45 (0.52; 11.47) | 3.45 (0.32; 37.33) |
| **Random intercept** | County | ***p <0.001*** | ***p= 0.024*** | ***p <0.001*** | ***P<0.001*** |

**Table I Multivariable analysis of factors associated with baseline infection levels.** Estimates were obtained by school level logistic and negative binomial regression analysis taking into account clustering within counties. Variables adjusted for in the final model are indicated in italic; variables significantly associated (p<0.05) are indicated in bold.

**3. Determinants of treatment coverage**

The difference in treatment coverage between the MDA rounds in years one and two was quantified using a school-level mixed effects logistic regression model with treatment coverage in years one and two as repeated outcome measure and county random intercepts. To investigate county characteristics associated with treatment coverage, county variables were combined into the five indicators education systems, health systems, logistics/infrastructure, economy and capacity to deliver health services using PCA (Table C). The associations of school treatment coverage with county characteristics were investigated separately for year one and two in 153 schools for which baseline and year three parasitological data were available using mixed effects logistic regression models with a random intercept for counties. In the univariable analysis, PCA scores were first included as linear terms and deviations from linear relationships were investigated by inclusion of quadratic terms. Multivariable models were fitted using a backwards approach, were first all indicator scores with univariable p-values <=0.05 were included (plus quadratic terms where relevant). Indicators with non-significant p-values (p>0.05) were subsequently removed starting with the highest p-value.

Within the 153 schools that form the basis of this analysis, the median reported treatment coverage in year one and two was of 93.2% (IQR 89.6-96.5%) and 94.6% (IQR 90.4-97.7), respectively. The treatment coverage in year two was significantly higher than in year one (OR 1.58, 95% CI 1.5-1.6, p<0.001). Treatment coverage by year and county, as well as the within county coverage heterogeneity, is shown in Fig D. In year one, the school treatment coverage varied markedly within counties, especially for counties with lower treatment coverage while less within county variation was observable in year two, where treatment coverage was generally higher. Results of univariable and multivariable logistic regression analysis of factors associated with treatment coverage are summarised in Table J. In both years higher treatment coverage was associated with higher scores for education systems (p<0.001, p=0.003).

|  | **Year 1** | | **Year 2** | |
| --- | --- | --- | --- | --- |
|  | **OR (95%CI)** | **p-value^1^** | **OR (95%CI)** | **p-value^1^** |
| **Univariable** | | | | |
| Education systems | **1.43 (1.22; 1.68)** | **<0.001** | **1.18 (1.08; 1.29)** | **0.003** |
| Education systems (squared term) | **1.15 (1.08; 1.23)** | **<0.001** | - |  |
| Health systems | 1.06 (0.90; 1.25) | 0.462 | 1.02 (0.93; 1.11) | 0.709 |
| Health service delivery | 1.15 (0.89; 1.49) | 0.288 | 1.07 (0.88; 1.15) | 0.350 |
| Logistics | 1.14 (0.91; 1.41) | 0.262 | 0.99 (0.88; 1.11) | 0.807 |
| Economy | 1.18 (0.92; 1.51) | 0.203 | 1.00 (0.88; 1.15) | 0.913 |
| **Multivariable** | | | | |
| Education systems | **1.43 (1.22; 1.68)** | **<0.001** | **1.18 (1.08; 1.29)** | **0.003** |
| Education systems (squared term) | **1.15 (1.08; 1.23)** | **<0.001** | - |  |
| Health systems | 0.98 (0.87; 1.10) | 0.748 | 1.05 (0.99; 1.11) | 0.100 |
| Health service delivery | 1.04 (0.86; 1.19) | 0.665 | 1.00 (0.91; 1.11) | 0.956 |
| Logistics | 1.10 (0.94; 1.29) | 0.240 | 1.05 (0.97; 1.14) | 0.261 |
| Economy | 1.01 (0.86; 1.19) | 0.869 | 0.98 (0.89; 1.07) | 0.605 |

^1^ based on likelihood ratio test

**Table J Results of univariable and multivariable logistic regression analysis of factors associated with treatment coverage.** The final models for year one and two were adjusted for education system scores, the year two model additionally included a quadratic term. Both models include a random intercept for counties.

| **A** |
| --- |
| **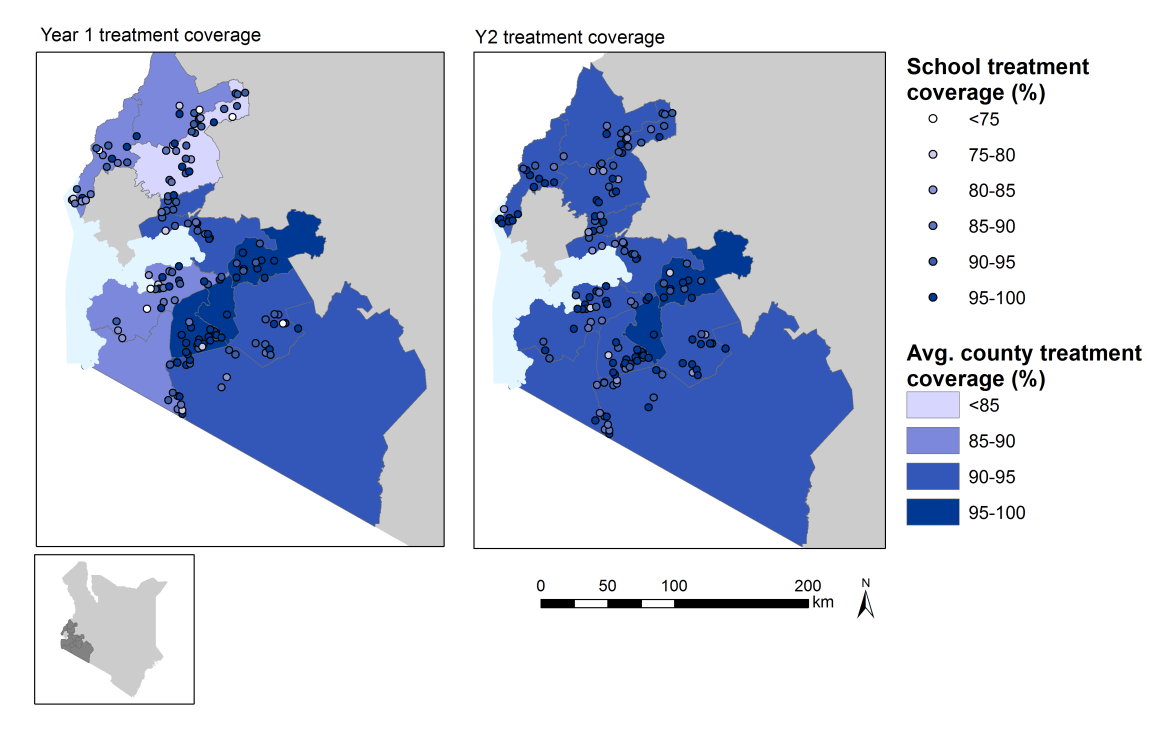** |
| **B** |
| 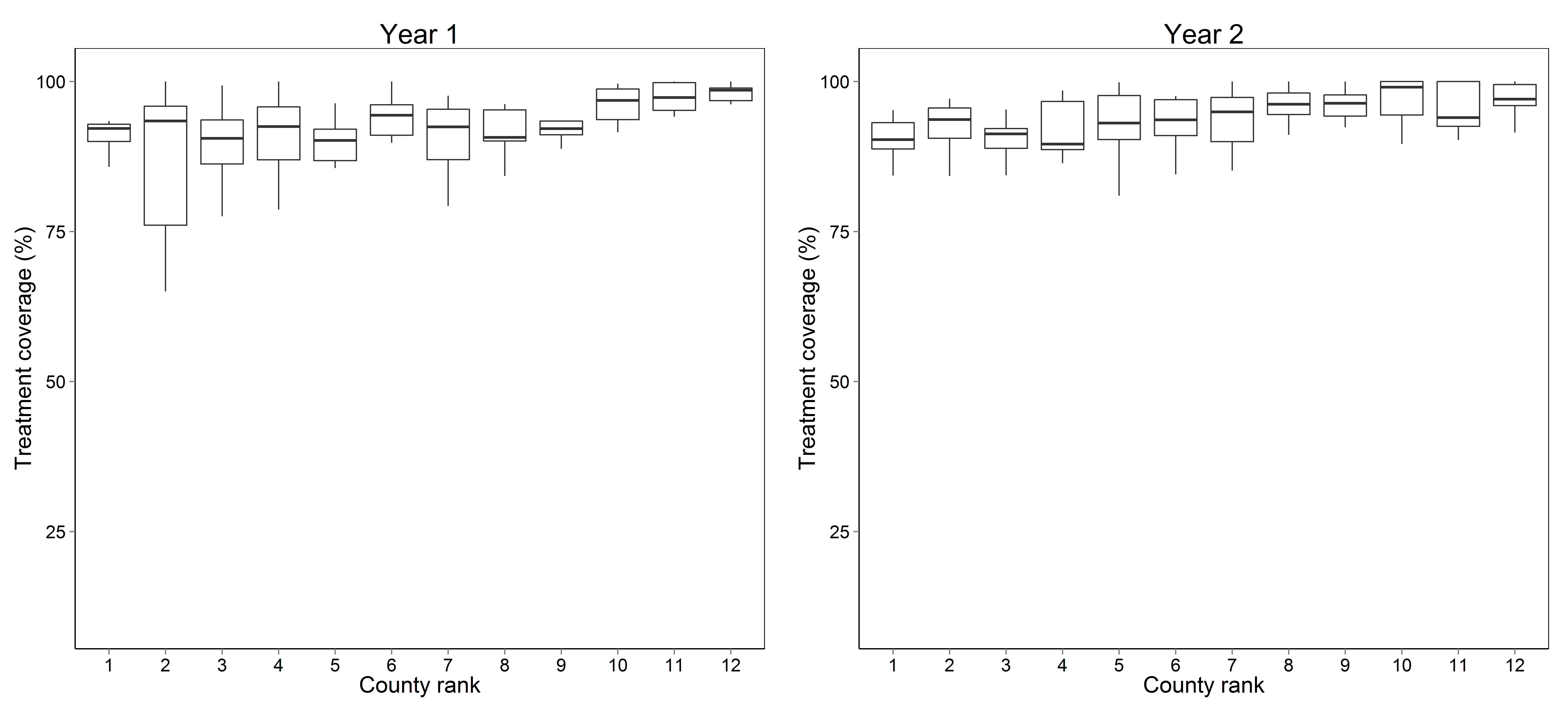 |

**Fig. D Treatment coverage in year one and two MDA delivery (A) and within county heterogeneity of treatment coverage (B).** The average county treatment coverage was calculated based on the 153 schools and counties were ranked lowest to highest according to average treatment coverage.

**References**

1. Brooker SJ, Nikolay B, Balabanova D, Pullan RL (2015) Global feasibility assessment of interrupting the transmission of soil-transmitted helminths: a statistical modelling study. Lancet Infect Dis 15: 941-950.

2. Health Policy Project, Kenya Ministry of Health. Kenya County Health Fact Sheets. (2014) Available at: <http://www.healthpolicyproject.com/>.

3. Kenya ICT Board. Kenya Open Data. (2014) Available at: https://opendata.go.ke/.

4. Africa Soil Information System. (2013) Available at: <http://www.africasoils.net/data/datasets>.

5. Consortium for Spatial Information. CGIAR. (2015) Available at: [www.cgiar-csi.org/](http://www.cgiar-csi.org/).

6. WorldPop project. (2013) Available at: <http://www.worldpop.org.uk/>.

7. The Kenya National Bureau of Statistics. Population and Housing Census. (2009) Available at: <http://www.knbs.or.ke/>.

8. Comission on Revenue Allocation (2013) County Budgets: 2013-2014.
